# Sevuparin in children with severe malaria: a phase I safety and dose-finding trial

**DOI:** 10.1038/s41467-026-78536-3

**Published:** 2026-10-21

**Authors:** Mike Chaponda, Jonathan Gwasupika, Roisin Connon, Patrick Tshilab Kabwit, Sam Miti, Nchafatso Obonyo, Diana M. Gibb, Thomas N. Williams, Nicholas P. J. Day, Arjen M. Dondorp, A. Sarah Walker, Elizabeth C. George, Kathryn Maitland

**Affiliations:** 1 National Health Research and Training Institute (NHRTI), Ndola, Zambia; 2 Saint Pauls Mission Hospital, Nchelenge, Zambia; 3 Innovative Clinical Trials Unit, Institute of Clinical Trials & Methodology, University College London, London, UK; 4 Kenya Medical Research Institute (KEMRI)/Wellcome Trust Research Programme, Kilifi, Kenya; 5 Faculty of Medicine, Institute of Global Health Innovation, Imperial College, London, UK; 6 Mahidol-Oxford Tropical Medicine Research Unit (MORU), Faculty of Medicine, Mahidol University, Bangkok, Thailand; 7 Centre for Tropical Medicine and Global Health, Nuffield Department of Medicine, University of Oxford, Oxford, UK

**Keywords:** Paediatric research, Outcomes research

## Abstract

Mortality rates for African children with severe malaria remain high. Sevuparin, a heparin-like compound, as an adjunctive treatment, may improve outcomes due to its effects on *Plasmodium falciparum*-infected erythrocytes. A Phase I dose-finding trial in Kenyan and Zambian children with severe malaria aimed to identify the maximum tolerated dose (MTD) of sevuparin, given as three infusions (at 0, 8 and 16 h post-enrolment) using the continual reassessment method (ISRCTN32271864). The primary endpoint was dose limiting toxicity (DLT) defined as Activated Partial Thromboplastin Time (APTT) > 95 seconds (2.5x upper limit of normal) across 1.5-6 mg/kg doses. Secondary endpoints were lactate clearance, parasite half-life, APTT at 24 h and grade 3-4 serious adverse events. Twenty-three children were enroled; 20 were included in the primary analysis, across 10 cohorts. A data monitoring committee followed model recommendations, leading to dose escalation from 1.5 mg/kg through all doses to 6 mg/kg when 2 DLTs occurred. During de-escalation, DLTs occurred at 5 mg/kg and 4 mg/kg. The final dose was therefore 3 mg/kg. There was 1 death (1/23 (4.3%)), 1 readmission and 1 grade 3 non-DLT adverse event: all judged unrelated to sevuparin. MTD of 3 mg/kg sevuparin in each of three infusions at 0, 8 and 16 h was identified for future investigation.

## Abstract (web summary)

This trial demonstrates the safety of sevuparin, a heparin-like compound, as an adjunctive therapy in severe malaria in African children and identified, using a continual reassessment method design, the optimal dose to investigate in future trials.

## Introduction

Malaria remains a key cause of paediatric hospital admission in sub-Saharan Africa and makes a substantial contribution to under 5-year mortality, estimated at ~600,000 annually^1,2^. In 2023, about 94% of *Plasmodium falciparum* malaria cases and 95% of global deaths occurred in the WHO African region^2^. Despite the widespread use of fast-acting artemisinin-based combination therapies, inpatient mortality for severe malaria remains unacceptably high at ~7–8%, as reported by Akech et al., 2020^3^ and SMAART Observational study ISRCTN14711763 (unpublished) and will not improve without better supportive treatments. Indeed, in children with the severest manifestation of cerebral malaria, namely deep unarousable coma, mortality of ~20-25% has largely remained unchanged over the decades^3,4^ or with the introduction of artesunate^5^. The most promising vaccines, RTS,S (3 doses plus booster) and R21, offered short-term protection against uncomplicated malaria^6^, but their effects on severe malaria and mortality have not yet been fully evaluated^7^. Clinical trials investigating the safety and efficacy of supportive treatments could fill existing gaps in the severe malaria treatment algorithm and substantially improve outcomes^8^. Yet, the paucity of clinical trial data is stark: only 33 clinical trials of supportive therapies have been conducted globally over the last 40 years, mostly single-centre Phase I or II trials involving few participants; none showed benefit^9^, and several were stopped early for harm^10^. This is despite the fact that, in the recent past, over a million African children were dying from malaria annually^11^.

*Plasmodium falciparum* parasite-infected erythrocytes (iRBC) have the unique ability to cytoadhere to both endothelia^12^ and to uninfected red cells (rosetting)^13^, and to cause sequestration of iRBC and RBC in the microvasculature, which is central to the pathology of severe malaria. Autopsy studies have shown intense sequestration in vital organs in children and adults who have died from malaria^14,15^, and most paediatric deaths from severe malaria in African hospitals occur soon after admission^5^. Supportive treatments that can reverse sequestration rapidly could therefore substantially improve survival in children admitted to hospital with severe malaria.

Heparin was previously investigated as an adjunctive treatment, hypothesised to reverse iRBC sequestration in vivo as it binds to *P. falciparum* erythrocyte membrane protein 1 (PfEMP1), the key parasite adhesion molecule mediating sequestration^16,17^. Furthermore, heparin blocks merozoite invasion into red blood cells in vitro^18^, a vital part of the malaria cycle of amplification of parasite load. However, clinical trials of heparin gave variable results, with some showing reduced mortality in children with severe *P. falciparum* malaria^19,20^, some showing no clinical improvements when given at low dose^21^ and others demonstrating potentially dangerous effects on coagulation^22^. Sevuparin is a low-anticoagulant heparin-like compound that is devoid of the negative effects of heparin on coagulation. It is a heparin derivative which is chemically modified so that the specific pentasaccharide domain responsible for high-affinity binding to antithrombin III is eliminated^23^. It therefore no longer includes the specific binding sequence for antithrombin, the main contributor to prolonged coagulation. As a result, sevuparin has no direct effect on factor Xa or thrombin, and its effect on activated partial thromboplastin time (APTT) is markedly reduced in comparison to standard dose heparin^24^. Like heparin, sevuparin binds to PfEMP1, reversing iRBC adhesion^23,25^ and rosetting, unblocking the microvasculature and restoring normal blood flow.

In a Phase I dose escalation trial in adults, sevuparin was shown to be safe and well-tolerated with minimal effects on APTT at doses of 1.5 mg/kg and 3 mg/kg every six hours, but a dose of 6.0 mg/kg led to higher APTT values^26^. The subsequent Phase II trial conducted in adults with uncomplicated *P. falciparum* malaria compared sevuparin 6-hourly intravenous infusions over 5 minutes versus standard treatment alone (control). There were minimal and non-clinically relevant changes in anti-Xa and prothrombin times and the international normalised ratios (INR) with sevuparin^26^. Increases in APTT were dose-dependent and appeared to follow the time-concentration curve for each sevuparin infusion. The 6-hour interval between infusions allowed for nearly full reversibility of APTT increases after each dose. No adverse effects were reported. Sevuparin has also been studied in children and adults for the treatment of acute vaso-occlusive crisis in patients with sickle cell disease (and has been granted Orphan Drug Designation and Rare Paediatric Disease Designation by the FDA for this indication). A Phase II trial in this population demonstrated that it was safe and well-tolerated, but it did not prevent the initiation of vaso-occlusive crises^27^.

We therefore hypothesised that in African children hospitalised with severe malaria, sevuparin would improve microcirculatory flow by reversing existing and preventing further parasite sequestration and hence reduce plasma lactate concentrations, a biomarker associated with poor outcomes directly linked to the degree of impaired perfusion^28^. However, to test this hypothesis, it was first essential to confirm the most appropriate sevuuparin dose in children, the primary objective of this Phase I trial. A secondary objective was to assess whether lactate clearance at 8 h could be a realistic and feasible primary endpoint for a subsequent Phase II trial.

## Results

**Fig. 1:**
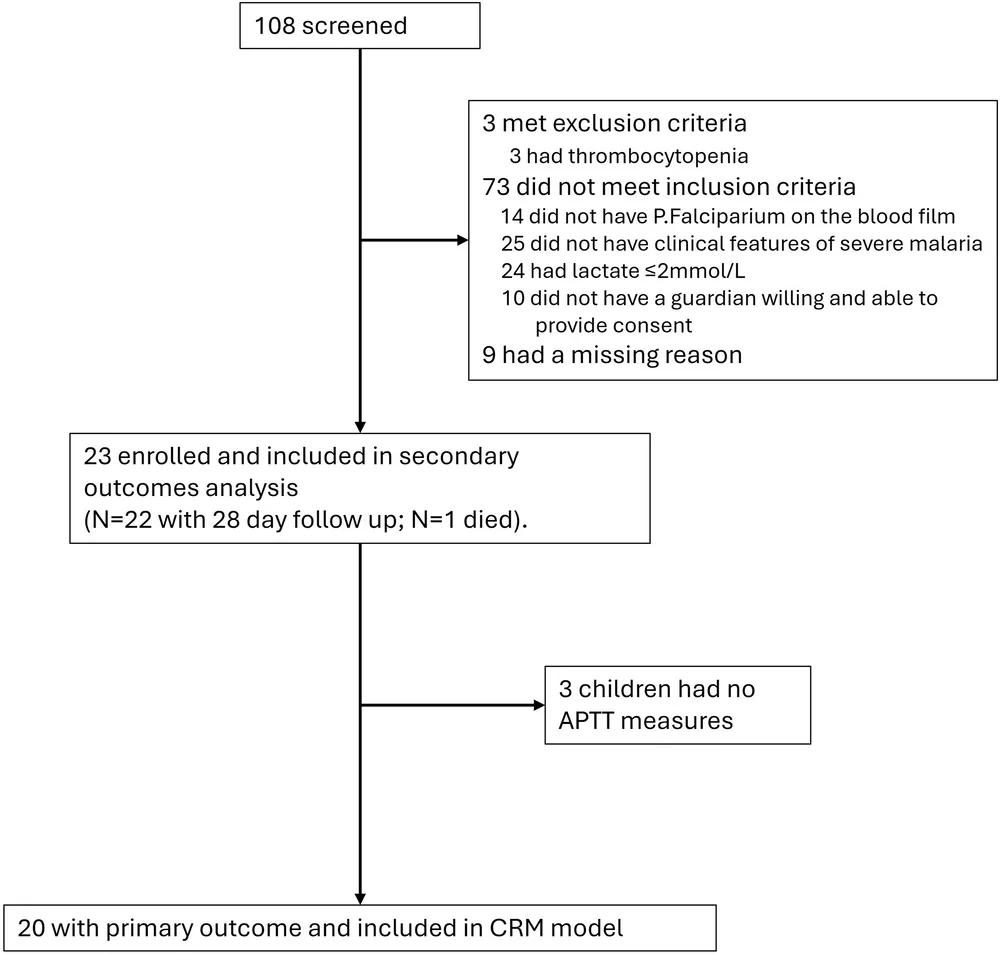
CONSORT diagram. CRM: continuous reassessment model.

Between 1st September 2022 and 5th March 2025, 23 children in Kenya and Zambia were enroled and followed up to Day 28 (Fig. 1). All 3 children enroled in Kenya were excluded from analyses as APTT measurements were not available and they therefore could not be included in the continual reassessment model. Baseline clinical and laboratory features are summarised in Table 1 (20 children included in the primary analysis, all recruited in Zambia), and Table S1 (all 23 children). In the 20 children included in the primary analysis, the median age was 35 months (IQR 24, 48) and 12 (60%) were female. 10 (50%) children had unrousable coma (cerebral malaria; Blantyre Coma Score≤2); median haemoglobin was 5.2 mg/dl (IQR 4.6, 6.3): 3 (15%) had severe malarial anaemia (defined as <4 g/dl); and 7 (35%) had deep ‘acidotic’ breathing. Median lactate was 3.5 mmol/l (IQR 2.6, 6.6).

**Table 1:** Baseline characteristics of the 20 children included in primary analysis

	N (%) or median (IQR)
Age (months)	35 (24, 48)
Aged <1 year	2 (10%)
Sex: Female	12 (60%)
Axillary temperature (°C)	37.5 (37.1, 38.3)
Blantyre Coma Score	
1	3 (15%)
2	7 (35%)
3	7 (35%)
4	2 (10%)
5	1 (5%)
Two or more seizures in last 24 h	8 (40%)
Did the seizures last >30 mins?	2 (25%)
Hemoglobinuria	2 (10%)
Neck stiffness	1 (5%)
Deep breathing	7 (35%)
Haemoglobin (g/dL)	5.2 (4.6, 6.3)
Haemoglobin<4 g/dl	3 (15%)
Lactate (mmol/L)	3.5 (2.6, 6.6)
Glucose (mmol/L)	6.3 (4.4, 8.6)
Parasite density (/µL)	174048 (48360, 432056)
APTT (seconds)	38.5 (36.4, 43.0)
Already received IV antimalarials	11 (55%)
Already received oral antimalarials	10 (50%)

N (%) or median (IQR). *APTT* Activated Partial Thromboplastin Time. All 20 children included in the primary analysis were recruited from Zambia.

### Adherence to dosage

**Fig. 2:**
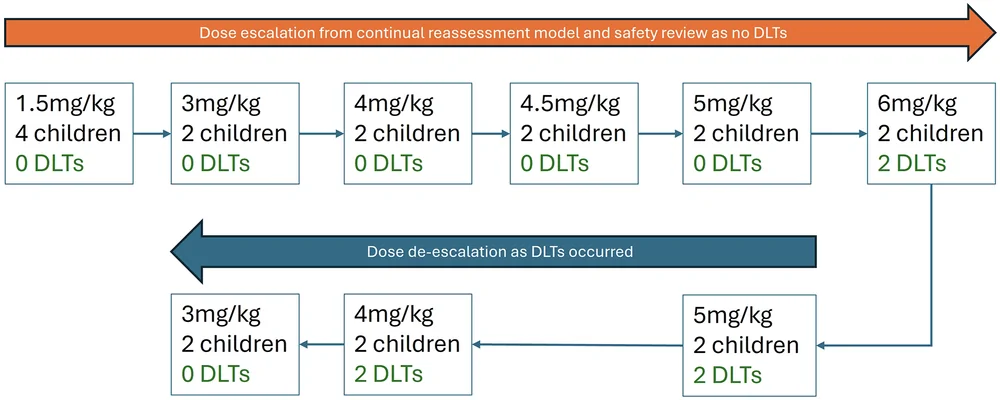
Dose escalations and deescalations in the trial. DLTs Dose-limiting toxicities.

Following the protocol, all children in the dose escalation cohorts adhered to the recommended dosage, or stopped subsequent dosages due to the development of dose-limiting toxicity (DLT), defined as Activated Partial Thromboplastin Time (APTT) > 95 s (2.5x upper limit of normal). As planned for the continual reassessment model, the first cohort of 4 children received each sevuparin infusion (given over 5 min) at 0, 8 and 16 h after enrolment starting at 1.5 mg/kg per dose; followed by an escalation through 3 mg/kg, 4 mg/kg, 4.5 mg/kg, 5 mg/kg and 6 mg/kg in cohorts of 2 children each following the model’s recommendations. Only at the 6 mg/kg dosage, after 14 children had been enroled, were two DLT noted. The Data Monitoring Committee (DMC) recommended a reduction to 5 mg/kg for the next cohort (*n* = 2) following the model’s recommendation. At the 5 mg/kg dose both children had a DLT; this was followed by a recommendation to de-escalate to 4 mg/kg for the next cohort (n = 2) (skipping the 4.5 mg/kg dose level) and both children at 4 mg/kg developed DLT. The DMC recommended de-escalation to 3 mg/kg for the final cohort (n = 2) following the model’s recommendation, and no DLT were reported (Fig. 2 and Table S2).

### Primary Endpoint

**Fig. 3:**
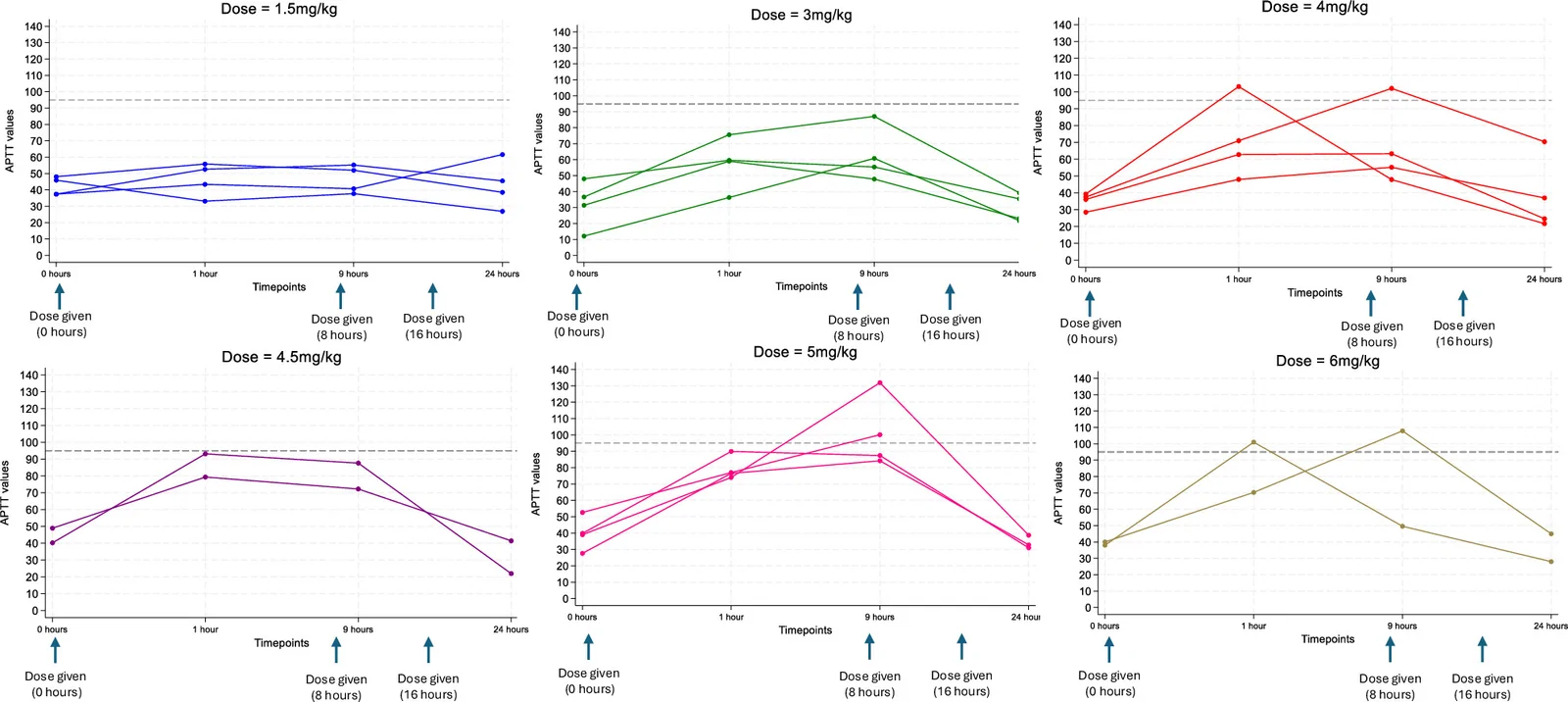
APTT levels in each child at each timepoint by dose level. Each plot represents a dose level. The dashed line at APTT = 95 defines a dose-limiting toxicity. If a child had an APTT > 95 then they did not receive subsequent doses of sevuparin. Source data are provided as a Source Data-SEVUSMART.xls file (https://doi.org/10.7910/DVN/6ZFNJ6).

The recorded APTT levels over 24 h for individual children at each dosage are presented in Fig. 3. Six (30%) children had DLT: 2 in each of the three doses of 4, 5 and 6 mg/kg. Most APTTs recorded as DLTs were in the range between >95 and <110 s. Only one child (who received 5 mg/kg) had an APTT > 110 s. DLT were recorded at 1 h (*n* = 2), and at 9 h (one hour post 2nd dose; *n* = 4); none were recorded at 24 h (8 h post 3rd dose). At 24 h, the overall mean APTT was 36 s (sd 13.2) (Table 2). There were no adverse clinical events related to the DLT, including no indication of any bleeding in any child.

**Table 2:** Outcomes and Adverse Events

Outcome	Dose level (mg/kg)							Total
	1.5	3	4	4.5	5	6		
	*N* = 4	*N* = 4	*N* = 4	*N* = 2	*N* = 4	*N* = 2	*N* = 20	
Efficacy outcomes								
Change in lactate at 8 hrs (mmol/L)^a^	−0.6 (1.6)	−0.3 (2.9)	−1.8 (1.8)	−0.7 (-)	−7.1 (2.5)	−1.1 (-)	−1.9 (3.1)	
Change in lactate at 24 hrs (mmol/L)^a^	1.0 (1.7)	−1.3 (2.2)	−1.8 (1.4)	−1.5 (-)	−7.4 (3.7)	−1.2 (-)	−1.9 (0.6)	
Parasite half-life (hours) (geometric mean)	-^b^	- ^b^	4.2 (*N* = 2)	11.1 (*N* = 1)	8.2 (*N* = 1)	5.0 (*N* = 1)	6.0 (3.5,10.4) ^c^	
Presence of mature infected erythrocytes at 8 h	0	0	0	0	0	0	0	
Presence of mature infected erythrocytes at 24 h	0	0	0	0	0	0	0	
Safety outcomes								
APTT at 24 hrs	43.1 (14.5)	29.9 (8.7)	38.4 (22.3)	31.7 (-)	34.2 ^d^ (-)	36.5 (-)	36.0 (13.2)	
Neurological sequalae at day 28	0	0	0	0	0	0	0	
Adverse events*								
Deaths	0	0	0	0	1	0	1	
Non-fatal SAES by day 28	0	0	1	0	0	0	1	
Adverse events grade 3 or 4 by day 28	0	0	2	0	3	2	7	

Arithmetic mean (sd) or N (%) presented unless specified otherwise. Standard deviation not shown when *N* < 4.

^a^One child died prior to the 24 h measurement of lactate (in the 5 mg/kg dose). Two children (1 in the 4.5 mg/kg dose and 1 in the 5 mg/kg dose) did not have a lactate measured within the window around 8 h.

^b^Not estimated as ≤2 measurements in each child.

^c^95% confidence interval presented around geometric mean.

^d^One child died prior to the 24 h measurement of APTT.

*A dose limiting toxicity of APTT > 2.5xULN was also a grade 3 adverse event and 2/2 events in dose level 4, 2/3 events in dose level 5 and 2/2 events in dose level 6 were dose-limiting toxicities. These were deemed possibly or probably related to sevuparin. The other adverse event at the dose of 5 mg/dg was a grade 3 clinical event (fever and cough), unlikely related to sevuparin. The non-fatal SAE was a hospital readmission due to anaemia and unlikely related to sevuparin. The death due to complicated malaria was judged unlikely related to sevuparin.

### Secondary endpoints

The overall change in lactate in the window around 8 h (5–12 h) was a mean reduction of 1.9 mmol/L (sd 3.1; measured on 18/20 children); at 24 h the decrease was also 1.9 mmol/L (sd 0.6; measured on 19/20 children). Parasite clearance half-life could only be estimated in 5/20 children since this required 3 measurements of slide parasitaemia; 10/15 children in whom this could not be calculated were only slide positive at baseline and 9 h and 5/15 children were slide positive only at baseline. In the 5 children in whom this could be calculated, the overall geometric mean parasite half-life was 6 h (95%CI 3.5-10.4 h). There were no mature infected erythrocytes (schizonts) seen on microscopy at 9 or 24 h.

### Safety endpoints and additional analyses

In the 20 children included in the primary analysis, there were 7 grade 3 adverse events of which 6 were the laboratory-defined DLTs without clinical complications described above. The additional grade 3 adverse event was a high fever and cough. There was also one non-fatal serious adverse event (hospital readmission for anaemia) and one death due to complications of malaria, judged unlikely to be related to sevuparin by the clinical team and on independent review. There were no neurological sequalae reported at the day 28 visit (Table 2).

An additional analysis of primary, secondary and safety endpoints in all children (including those without APTT measures) gave similar results (Table S3). There was only one additional adverse event, which was a hospital readmission for convulsions.

In children with lactate measurements in the primary analysis population, 5/18 (28%) had lactate ≤2 mmol/L in the window around 8 h, and 7/19 (37%) at 24 h (Fig. S1).

## Discussion

Through this small Phase I single-arm trial, we have confirmed a paediatric dose, and ascertained further evidence on the safety of sevuparin given as an adjunctive therapy alongside intravenous artesunate. All APTT dose-limiting toxicities, also reported as grade 3 adverse events, were clinically silent. In the primary analysis population, the only other adverse events reported were one grade 3 fever and cough and two serious adverse events (one death and one readmission), all judged unlikely to be related to sevuparin. We established, using the continual reassessment method, the maximum tolerated dose of sevuparin of 3 mg/kg in children hospitalised with severe malaria. This is similar to the dose recommended for future investigation in the adult trial Phase I trial^26^. A secondary objective was to investigate lactate clearance as a potential endpoint for future trials: 28% of children demonstrated early lactate clearance (to <2 mmol/L by 8 h) and 75% had cleared their parasitaemia by 24 h. As the trial was designed as a dose-finding study, there was no comparator arm for comparison. However, of note, the observed overall mortality rate in the 23 children enrolled (4%, 95% CI 0.1–21%) was much lower than expected given that 50% of the children in the trial had the severest clinical presentation of unrousable coma (cerebral malaria) and metabolic acidosis (lactate >2 mmol/l). We used the PET score^29^ to predict expected mortality, a score developed from the FEAST trial data and validated in external data sets to assess risk of mortality from baseline clinical variables. The majority (19/20) of the children in this study had scores ≥5, putting them at ≥10% risk of mortality (Table S4). Furthermore, mortality rates to Day 28 in children included in the SMAART observational study (conducted 2022-2024) who presented in coma complicated by metabolic acidosis (lactate >2 mmol/l) were 24/70 (34%) in Nchelenge, Zambia and 4/19 (21%) in Kilifi, Kenya (personal communication). The observed low mortality may also be due to additional clinical monitoring given to children in this clinical trial; however, the same clinical teams managed children in both the SMAART observational study and this Phase I trial. Further, in the previous AQUAMAT trial, mortality rates remained high in children with coma in both artesunate and quinine arms (18% vs 21%)^5^ despite the provision of monitoring and close clinical management within a Phase III trial.

One of the strengths of the trial is the excellent adherence to dosing strategy (100%); in those with valid measures of APTT (20/23) there was 100% compliance with DLT stopping rules and similar completeness in follow-up and ascertainment of survival status. Operationally, the conduct of the trial teams through planned recruitment pauses following completion of each dosage cohort, timely analysis to update the model's recommendation for the next dosage and rapid review by the independent DMC was of high quality and carried out closely following the protocol.

Trial limitations included the paucity of laboratory measures of severity at baseline (including biomarkers assessing renal function and quantification of parasite biomass (*Pf*HRP2 measurement) and less frequent measurements of parasitaemia on blood films, both a consequence of aiming to minimise additional blood draws given the need to take samples to assay sevuparin pharmacokinetics given the dose-finding primary objective of the trial. In particular, the blood film sample timepoints were aligned to the clinical sampling schedule (with the first post-baseline sample taken at 9 h) rather than to the Worldwide Antimalarial Resistance Network (WWARN) parasite clearance estimator, which recommends sampling every 4 h. We were therefore not able to calculate parasite clearance on all children (as this requires at least 3 time points with parasitaemia present on the slide). Owing to the rapid parasite clearance afforded by artesunate, any additional benefit of sevuparin on clearance of early ring-stage parasites, as demonstrated in the TSM02 study in uncomplicated *P. falciparum* malaria in adults^26^, could not be seen clearly. Future investigation of sevuparin should incorporate 4-hourly monitoring of parasitaemia as well as parasite staging. We did not find evidence of mature parasites (schizonts) at the two time points (both ~1 h post-infusion), which would have provided further evidence for de-sequestration by sevuparin. However, the most likely explanation is that schizonts would be cleared very rapidly by the spleen. Future investigation of circulating mature parasites should include assessment during the time of sevuparin infusions. Finally, we were unable to record changes in sublingual circulation as a secondary endpoint (as planned in the protocol) as the CytoCam (Braedius Medical BV®) machine was non-functional by the time the study was initiated (delayed due to COVID-19). Investigation of sublingual circulation to provide supportive physiological/mechanistic data should be considered in future studies.

Overall, these data provide support for a future multi-site randomised controlled Phase II trial, with nested pathophysiological and pharmacokinetic studies. A future trial could generate supportive mechanistic data investigating the magnitude of de-sequestration and invasion-blocking by sevuparin and enable comparison to a control group. This would also provide experimental data to assess associations with laboratory and clinical outcomes to determine expected or unexpected biological mechanisms. For example, parasite adhesion properties have been shown to vary between *P. falciparum* isolates from different regions, with some showing high levels of rosetting^30^, while others show high levels of organ-specific cytoadhesion, e.g., to brain endothelial cells^31^. With an identified maximum tolerated dose, future mechanistic studies alongside a randomised controlled trial would help demonstrate the impact on expected mechanisms and clinical outcomes from this adjunctive therapy.

We have identified the maximum tolerated dose of 3 mg/kg and confirmed the safety of sevuparin as a supportive treatment for future investigation in children with severe malaria.

## Methods

### Ethics statement

Ethical approval was obtained from Imperial College Research Ethics Committee (ICREC) on 13th July 2021 (protocol number 18IC4513), Kenya Scientific and Ethics Review Unit (SERU), Nairobi Kenya on 25th February 2019 (protocol number GCMR-C/127/3744), Imperial College Research Ethics Committee (ICREC) on 13th July 2021 (protocol number 18IC4513) and the Tropical Diseases Research Centre Ethics Review Committee Ndola, Zambia and National Health Research Ethics Board, Lusaka, Zambia on 24th July 2023 (protocol number NHREB001/24/07/2023).

### Trial design

This was a phase I open-label safety and dose-finding trial with a primary objective to identify the maximum tolerated dose (MTD) of intravenous sevuparin as an adjunctive therapy in children with severe malaria given as three infusions at 0, 8 and 16 h. A secondary objective was to assess whether lactate clearance at 8 h is a realistic and feasible primary endpoint for a subsequent Phase II trial.

The trial used the continual reassessment method to identify the MTD. The continual reassessment method starts with an a priori dose-toxicity curve, reflecting the probability of experiencing a DLT event at the dose received. For this trial we defined a DLT as an APTT > 2.5 times the upper limit of normal (ULN) (grade 3 following the Common Toxicity Criteria v5.0^32^) 1 h after each dose. The dose-toxicity curve was then re-fitted to the data after each cohort of 2 children was enrolled, based on their observed dose and whether they had a dose-limiting toxicity event. After the first 4 children, each subsequent cohort was assigned the next highest dose, until the estimated risk of toxicity was just below or at the target toxicity level, designated as the MTD. The dose-limiting toxicity, a priori dose-toxicity curve and chosen target toxicity rate (15%) used in this trial were based upon data shared by the investigators from the adult sevuparin studies in Thailand^26^. We planned to enrol 20 children since this would allow sufficient data on safety to be generated across a range of doses to identify the MTD from a more informed model relating dose to toxicity events (denoted the ‘dose- toxicity’ curve) than that available a priori based on published data from adult studies.

The protocol for this study has been previously published^33^. Trial registration ISRCTN32271864 (registered on 28/07/2021) and PACTR202007890194806 (registered on 20/07/2020). The Statistical Analysis Plan is available on the ISRCTN registration.

### Study population

Children hospitalised with severe malaria were enrolled at Kilifi County Hospital, Kilifi, Kenya and, following a protocol amendment, at Saint Pauls Hospital, Nchelenge Luapula Province, Zambia. Both male and female children were eligible for enrolment if they were aged 3 months to 12 years on the day of admission with a slide positive for *P. falciparum* malaria in addition to all of the following: clinical evidence of severe malaria (any one of the following: impaired consciousness (defined as coma (inability to localise painful stimulus) or prostration (inability to sit unsupported for those above 6 months) or deep breathing), plus a lactate level of >2 mmol/L, and a guardian or parent willing and able to provide consent. The specific severity criteria included were selected from current WHO definitions of severe malaria, with the goal of identifying children with higher parasite sequestration. Anaemia was therefore not included per se. Acute kidney injury was not included as neither creatinine or urea were measured at baseline to reduce blood draw, given the focus of the trial on sevuparin dosing. Lactate >2 mmol/L was required in all children to ensure we could address our secondary objective of identifying whether lactate clearance was a potential trial endpoint. Children with clinical evidence or a history of a bleeding/coagulation disorder, a comorbidity which clinicians believed had a significant risk of poor outcome, e.g. malignancy, end-stage renal failure, major cardiac condition or severe thrombocytopenia (platelet count <25 ×10^9^/L), were excluded from the trial.

### Study medication

Sevuparin, donated for the trial by the biotech startup Modus Therapeutics, Stockholm Sweden, has been approved for clinical research in Europe and America and remains an investigational drug that is currently in clinical development. The drug product, sevuparin 150 mg/mL solution for intravenous (IV) infusion, was formulated in a 0.015 M phosphate buffer at a pH of 7.0 and was stored in a refrigerator at 2–8° celsius and protected from light. The solution for administration was prepared in a syringe (diluted in saline) and kept refrigerated and used within 24 h.

The trial was open label, and clinicians and staff administering sevuparin were aware of the dosage. Laboratory staff performing APTT levels were blind to drug dosage level. Children received sevuparin as three infusions (over 5 min) at 0, 8 and 16 h after study enrolment. The initial participants (two ‘cohorts’ of 2 children each, i.e., 4 children in total) received a dose of 1.5 mg/kg/dose with the plan to escalate up to the next highest dose for each cohort of 2 children up to a maximum of 6.0 mg/kg/dose following the recommendations of the continual reassessment model and the Data Monitoring Committee (Fig. S2).

### Outcome measures

The primary outcome was development of a DLT defined as APTT > 2.5 x ULN (grade 3 following the Common Toxicity Criteria v5.0^32^) translating to > 95 s (assuming a normal range of 21–38 s) 1 h post sevuparin dose. Secondary outcomes included change in lactate from 0 to 8 h; presence of mature infected erythrocytes on the blood films at 8 and 24 h, and parasite clearance time. Safety endpoints included APTT 24 h post-enrolment (absolute level and grade), development of abnormalities of coagulation indices (prothrombin) (Grade ≥2), neurological sequelae through day 28, mortality through day 28, serious adverse events and Grade 3/4 adverse events through day 28.

### Study screening and consent to participate

Children admitted to the paediatric wards of in Kilifi County and St Pauls Hospital, Nchelenge in Kenya and Zambia, respectively, with suspected malaria and either coma or deep breathing (defining severe malaria for this Phase I study) were screened by the paediatric triage/admission team for eligibility. When prior written consent from parents or legal guardians could not be obtained, ethics committees approved verbal assent with delayed written informed consent as soon as practical^34^. Otherwise, written informed consent was obtained from parents or guardians before enrolment.

### Study procedures and follow-up

The clinical assessments, baseline characteristics (including sex, but not gender of participants, which was determined based on self-report by parents) and blood tests taken at admission and during the trial included standard-of-care and research bloods were recorded on standardised case report forms. These included admission and serial assessment of full blood count. Lactate (StatStrip® Lactate Metre, Nova Biomedical) was measured at admission, within a window of 5 to 12 h and within 22 to 26 h. Malaria parasitaemia was assessed by blood film microscopy at admission and at 9 h, 24 h and in 12 h intervals until 72 h or until a negative slide. APTT was measured (by the Sysmex semiautomated blood coagulation analyser (CA-104)) at admission (pre dose) and at 1 h and 9 h and 24 h. Samples were stored for future pharmacokinetic assays. Vital signs were clinically reviewed at 1, 2, 4, 8 and 24 h and then bi-daily until hospital discharge. Children received parenteral artesunate for at least 3 doses and then followed by an oral course of artemether-lumefantrine per WHO guidelines^35^. All trial patients received intravenous antibiotics. Intravenous maintenance fluids were given at a rate of 4 ml/kg/hour until the child was able to drink and retain oral fluids. Antipyretics, anticonvulsants and treatment for hypoglycaemia and other treatments were given according to nationally agreed protocols. Transfusions were provided for children with Hb <4 g/dl (or Hb <6 g/dl and respiratory distress (suspected Kussmaul's ‘acidotic’ breathing). Any child developing an APTT > 95 seconds (2.5xULN; grade 3 toxicity) did not receive further doses of sevuparin but continued to be followed up. Children were reviewed daily until discharge and followed up at day 7 and day 28. Children lost to follow-up before day 28 were traced for vital status using locator data (permission requested for this data within consent). Parents received compensation for transport and out-of-pocket expenses to attend follow-up.

### Data management and statistical analysis

Management and outcome data were collected on case report forms. This included bedside observations for clinical parameters, administration of sevuparin, number of transfusions, use of drugs (specifically anticonvulsants, paracetamol, and antibiotics), malaria slide readings, developmental assessment at 28 days, serious adverse events, and date of discharge or in-hospital death.

Mean and standard deviation (SD) were used to estimate change in lactate from baseline at 8 and 24 h, as well levels of APTT at 24 h, overall and by dose level. The lactate measurements were taken within a window around each timepoint, namely 5-12 h for the 8-h measurement and 22–26 h for the 24-h measurement. Data were not disaggregated for sex. Geometric mean and 95% confidence intervals were used to describe parasite clearance within 72 h, estimated using WWARN parasite estimator^36^. The R package bcrm was used to run the continual reassessment model^37^. The dose levels used in the trial were 1.5 mg/kg, 3 mg/kg, 4 mg/kg, 4.5 mg/kg, 5 mg/kg and 6 mg/kg with a-priori risks of a DLT of 0.01, 0.07, 0.1, 0.15, 0.2 and 0.35 respectively. The functional form of the a priori curve was a power model with Gamma(1,1). The model was set to have no dose skipping during escalation but no restriction during de-escalation. After the first 4 children were dosed at 1.5 mg/kg, subsequent children were enrolled in cohorts of 2. After each cohort, recruitment was paused, and the DMC reviewed accumulating data on baseline characteristics, sevuparin dosing and APTT levels, any serious adverse events and the model’s recommendation for the next dose level. The DMC then gave a recommendation to the site to continue at the chosen dose level.

### Reporting summary

Further information on research design is available in the Nature Portfolio Reporting Summary linked to this article.

## Supplementary information

Supplementary information

Reporting Summary

Transparent Peer Review file

## Source data

Source Data

## Data Availability

Data are available through the online repository for KEMRI–Wellcome Trust Research Programme: Harvard Dataverse. Individual-level data, excluding personal identifiers, will be made available in accordance with an application to the Data Governance Committee, which meets monthly (dgc@kemri-wellcome.org). There are no timing restrictions on the availability of data. Source data for figures are provided with this paper. Source data are provided with this paper.
