# Supplementary material for: Sevuparin in children with severe malaria: a phase I safety and dose-finding trial
Source: Nat Commun. 2026 Oct 21;17:10693. doi: 10.1038/s41467-026-78536-3 (PMC13686957; doi:10.1038/s41467-026-78536-3)
Supplement: Supplementary file 1 [file 41467_2026_78536_MOESM1_ESM.pdf]

Supplementary Information

Contents

Figure S1: Trial schema. 2
Figure S2: Spaghetti plot of lactate values over time 3
Table S1: Schedule of Assessments 4
Table S2: Baseline characteristics of all children enrolled. 5
Table S3: Decisions made by the Data Monitoring Committee 6
Table S4: Outcomes in all children enrolled 7
Table S5: CONSORT Checklist 8
Trial Protocol and Statistical Analysis Plan 11

Figure S1: Trial schema.

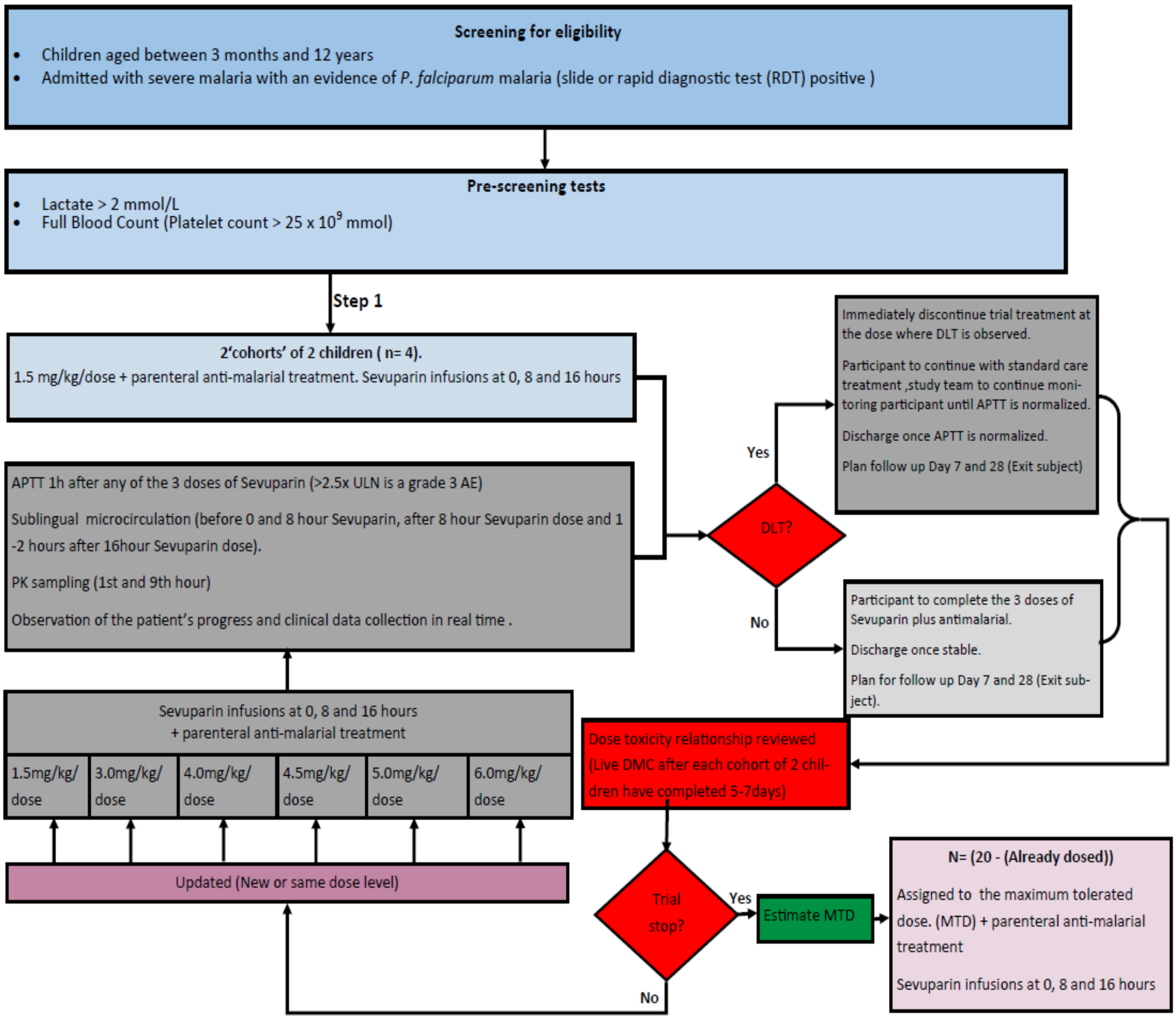

This figure shows a flow diagram of the trial to demonstrate the screening, enrolment and trial processes including the review after each cohort of 2 for a DLT based on levels of APTT and for the continual reassessment model and DMC to give a recommendation to continue or change the dose or to stop the trial.

Figure S2: Spaghetti plot of lactate values over time

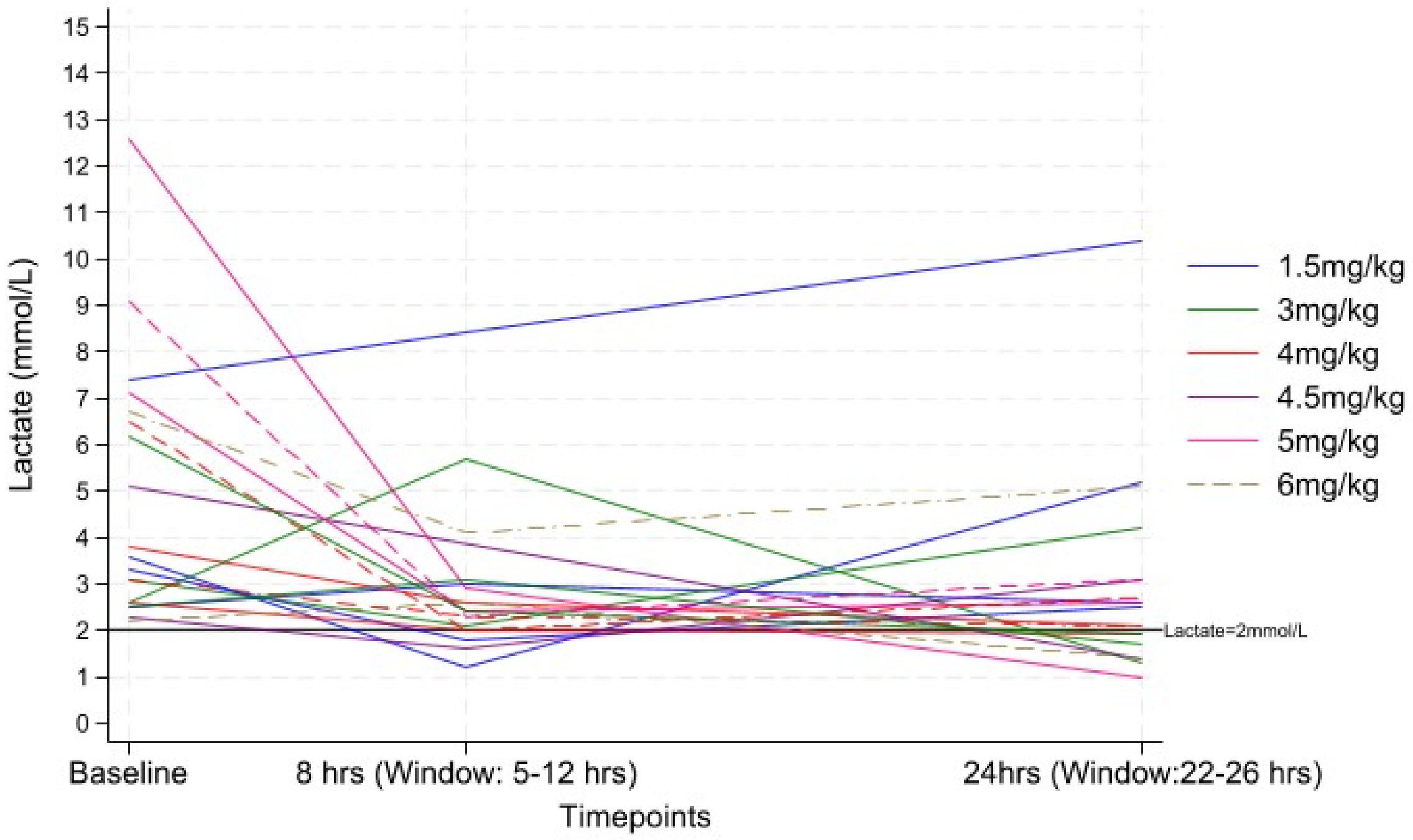

Each line represents 1 child receiving sevuparin at that dose. The line at lactate=2mmol/L was the lower limit of lactate for eligibility. Source data are provided as a Source Data file.

Table S1: Schedule of Assessments

	Assessment Time										
**Procedure**	**Adm**	**1h**	**2h**	**4h**	**8h**	**9h**	**17h**	**24h**	**Bi daily to discharge**	**Day 7**	**Day 28**
Clinical assessment	X	X	X	X	X			X	X	X	X
ECG continuous to 24 hours (Kilifi)	X							X			
APTT and coagulation tests (ACT) (1.7ml)	X	X 1 hr post dose				X 1 hr post dose	X 1 hr post dos e	X			
Lactate (point of care)	X	X				X		X			
Standard clinical test non-research	X							X		X	
Malaria slides	X					X		X	(36,48, 72 hrs)	X	X
Admission plasma **	X					X					X
PK sample		X				X					
Neuro-developmental assessment	X										X

* Standard- non research clinical tests full blood count will be done at admission and at 24 hours and Day 7 (Full blood count only).

Malaria slide and morphology will be done at 0, 9, 17, 24, 36, 48 hours (and 72 hours if in hospital); at follow up (Day 7 and 28)

**For quantitative plasma HRP2 assessment,

Table S2: Baseline characteristics of all children enrolled.

Characteristic	N=23
Site	
Kilifi	3 (13%)
Nchelenge	20 (87%)
Age (months)	35.0 (21.0, 54.0)
Aged <1 years old	3 (13.0%)
Gender - Female	13 (56.5%)
Axillary temperature (°C)	37.6 (36.9, 38.4)
Blantyre Coma Score	
1	4 (17.4%)
2	8 (34.8%)
3	7 (30.4%)
4	3 (13.0%)
5	1 (4.3%)
Two or more seizures within 24 hours	10 (43.5%)
Did seizures last >30mins	4 (40.0%)
Haemoglobinuria	2 (9.1%)
Neck stiffness	1 (4.3%)
Deep breathing	10 (43%)
Haemoglobin (g/dL)	5.5 (4.6, 6.7)
Haemoglobin <4g/dl	4 (17.4%)
Lactate (mmols/L)	3.6 (2.6, 6.5)
Glucose (mmol/L)	6.4 (4.4, 9.7)
Parasite density (/μL)	200688 (74062, 356684)
APTT (seconds) (N=20)	38.5 (36.4, 43.0)
Already received IV antimalarials	13 (56.5%)
Already received oral antimalarials	11 (47.8%)

Table S3: Decisions made by the Data Monitoring Committee

Dose (mg/kg)	Number enrolled	Number of dose limiting toxicities*	Decision
1.5	4	0	Escalate to 3mg/kg
3	2	0	Escalate to 4mg/kg
4	2	0	Escalate to 4.5 mg/kg
4.5	2	0	Escalate to 5mg/kg
5	2	0	Escalate to 6mg/kg
6	2	2	De-escalate to 5mg/kg
5	2	2	De-escalate to 4mg/kg
4	2	2	De-escalate to 3mg/kg
3	2	0	Enrolment completed

*Dose limiting toxicity defined by Activated partial thromboplastin time (APTT) >2.5 times the Upper Limit of Normal (ULN)

NB: Each decision was made after considering the Continual Reassessment model's recommendation.

Table S4: Outcomes in all children enrolled

Outcome	Dose level (mg/kg)						Total
	1.5	3	4	4.5	5	6	
	N=7	N=4	N=4	N=2	N=4	N=2	N=23
Efficacy outcomes							
Change in lactate at 8 hrs (mmol/L) [a]	-1.6 (1.9)	-0.3 (2.9)	-1.8 (1.8)	-0.7 (-)	-7.1 (2.5)	-1.1 (-)	-2.1 (2.9)
Change in lactate at 24 hrs (mmol/L) [a]	-0.4 (2.5)	-1.3 (2.2)	-1.8 (1.4)	-1.5 (-)	-7.4 (3.7)	-1.2 (-)	-1.9 (3.1)
Parasite half-life (hours) (geometric mean)	- [b]	- [b]	4.2 (N=2)	11.1 (N=1)	8.2 (N=1)	5.0 (N=1)	6.0 (3.5,10.4) [c]
Presence of mature infected erythrocytes at 8 hours	0	0	0	0	0	0	0
Presence of mature infected erythrocytes at 24 hours	0	0	0	0	0	0	0
Safety outcomes							
Neurological sequalae at day 28	0	0	0	0	0	0	0
Adverse events*							
Deaths	0	0	0	0	1	0	1
Non-fatal SAES by day 28	1	0	1	0	0	0	2
Adverse events grade 3 or 4 by day 28	0	0	2	0	3	2	7

NB: APTT was not measured in the three children included this analysis (compared to the main analysis) and so is not presented here.

Arithmetic mean (sd) or N (%) presented unless specified otherwise. Standard deviation not shown when N<4.

[a] One child died prior to the 24 hour measurement of lactate (in the 5mg/kg dose).

[b] Not estimated as ≤2 measurements in each child

[c] 95% Confidence interval presented around geometric mean

*A dose limiting toxicity of APTT>2.5xULN was also a grade 3 adverse event and 2/2 events in dose level 4, 2/3 events in dose level 5 and 2/2 events in dose level 6 were dose limiting toxicities. These were deemed possibly or probably related to Sevuparin. The other adverse event in dose level 5 was a grade 3 clinical event (fever), unlikely related to Sevuparin. The non-fatal SAE in dose level 4 was a hospital readmission due to anaemia and unlikely related to Sevuparin; and in dose level 1.5 was a hospital readmission due to convulsions and unlikely related to Sevuparin. The death was deemed unlikely related to Sevuparin and cause of death was complicated malaria.

Table S5: CONSORT Checklist

Section/topic	No	CONSORT 2025 checklist item description	Reported on page no.
**Title and abstract**			
Title and structured abstract	1a	Identification as a randomised trial	1
	1b	Structured summary of the trial design, methods, results, and conclusions	
**Open science**			
Trial registration	2	Name of trial registry, identifying number (with URL) and date of registration	2,9
Protocol and statistical analysis plan	3	Where the trial protocol and statistical analysis plan can be accessed	9
Data sharing	4	Where and how the individual de-identified participant data (including data dictionary), statistical code and any other materials can be accessed	11
Funding and conflicts of interest	5a	Sources of funding and other support (eg, supply of drugs), and role of funders in the design, conduct, analysis and reporting of the trial	1,12
	5b	Financial and other conflicts of interest of the manuscript authors	12
**Introduction**			
Background and rationale	6	Scientific background and rationale	3-4
Objectives	7	Specific objectives related to benefits and harms	4
**Methods**			
Patient and public involvement	8	Details of patient or public involvement in the design, conduct and reporting of the trial	n/a
Trial design	9	Description of trial design including type of trial (eg, parallel group, crossover), allocation ratio, and framework (eg, superiority, equivalence, non-inferiority, exploratory)	8
Changes to trial protocol	10	Important changes to the trial after it commenced including any outcomes or analyses that were not prespecified, with reason	9
Trial setting	11	Settings (eg, community, hospital) and locations (eg, countries, sites) where the trial was conducted	9
Eligibility criteria	12a	Eligibility criteria for participants	9
	12b	If applicable, eligibility criteria for sites and for individuals delivering the interventions (eg, surgeons, physiotherapists)	
Intervention and comparator	13	Intervention and comparator with sufficient details to allow replication. If relevant, where additional materials describing the intervention and comparator (eg, intervention manual) can be accessed	9

Outcomes	14	Prespecified primary and secondary outcomes, including the specific measurement variable (eg, systolic blood pressure), analysis metric (eg, change from baseline, final value, time to event), method of aggregation (eg, median, proportion), and time point for each outcome	10
Harms	15	How harms were defined and assessed (eg, systematically, non-systematically)	10
Sample size	16a	How sample size was determined, including all assumptions supporting the sample size calculation	9
	16b	Explanation of any interim analyses and stopping guidelines	10
Randomisation:			
Sequence generation	17a	Who generated the random allocation sequence and the method used	n/a
	17b	Type of randomisation and details of any restriction (eg, stratification, blocking and block size)	Fig S1
			**Reported on page no.**
Allocation concealment mechanism	18	Mechanism used to implement the random allocation sequence (eg, central computer/telephone; sequentially numbered, opaque, sealed containers), describing any steps to conceal the sequence until interventions were assigned	n/a
Implementation	19	Whether the personnel who enrolled and those who assigned participants to the interventions had access to the random allocation sequence	n/a
Blinding	20a	Who was blinded after assignment to interventions (eg, participants, care providers, outcome assessors, data analysts)	9
	20b	If blinded, how blinding was achieved and description of the similarity of interventions	
Statistical methods	21a	Statistical methods used to compare groups for primary and secondary outcomes, including harms	11
	21b	Definition of who is included in each analysis (eg, all randomised participants), and in which group	4
	21c	How missing data were handled in the analysis	n/a
	21d	Methods for any additional analyses (eg, subgroup and sensitivity analyses), distinguishing prespecified from post hoc	none
**Results**			
Participant flow, including flow diagram	22a	For each group, the numbers of participants who were randomly assigned, received intended intervention, and were analysed for the primary outcome	4
	22b	For each group, losses and exclusions after randomisation, together with reasons	Fig1 Consort
Recruitment	23a	Dates defining the periods of recruitment and follow-up for outcomes of benefits and harms	4
	23b	If relevant, why the trial ended or was stopped	n/q
Intervention and comparator delivery	24a	Intervention and comparator as they were actually administered (eg, where appropriate, who delivered the intervention/comparator, how participants adhered, whether they were delivered as intended (fidelity))	5
	24b	Concomitant care received during the trial for each group	10
Baseline data	25	A table showing baseline demographic and clinical characteristics for each group	Table 1, S1

Numbers analysed, outcomes and estimation	26	For each primary and secondary outcome, by group:<br>● the number of participants included in the analysis<br>● the number of participants with available data at the outcome time point<br>● result for each group, and the estimated effect size and its precision (such as 95% confidence interval)<br>● for binary outcomes, presentation of both absolute and relative effect size	5
Harms	27	All harms or unintended events in each group	6 Table 2
Ancillary analyses	28	Any other analyses performed, including subgroup and sensitivity analyses, distinguishing pre-specified from post hoc	n/a
**Discussion**			
Interpretation	29	Interpretation consistent with results, balancing benefits and harms, and considering other relevant evidence	6-7
Limitations	30	Trial limitations, addressing sources of potential bias, imprecision, generalisability, and, if relevant, multiplicity of analyses	7

Citation: Hopewell S, Chan AW, Collins GS, Hróbjartsson A, Moher D, Schulz KF, et al. CONSORT 2025 Statement: updated guideline for reporting randomised trials. BMJ. 2025; 388:e081123. https://dx.doi.org/10.1136/bmj-2024-081123
© 2025 Hopewell et al. This is an Open Access article distributed under the terms of the Creative Commons Attribution License (https://creativecommons.org/licenses/by/4.0/), which permits unrestricted use, distribution, and reproduction in any medium, provided the original work is properly cited.

*We strongly recommend reading this statement in conjunction with the CONSORT 2025 Explanation and Elaboration and/or the CONSORT 2025 Expanded Checklist for important clarifications on all the items. We also recommend reading relevant CONSORT extensions. See www.consort-spirit.org.

Trial Protocol and Statistical Analysis Plan

Sponsors

Funders

Sevuparin as a potential adjunctive therapy in children with severe malaria:

Phase I Safety and Dose finding Trial

(SEVUSMART trial)

**Protocol version:**	2.1
**Protocol date:**	28th February 2023
**ICREC number:**	**18IC4513**
**SERU Number:**	KEMRI/SERU/CGMR-C/127/3744
**Trial Sponsor:**	Imperial College London
**Sponsor reference:**	**P71159**
**PACTR number:**	**202007890194806**.
Trial Funder	Wellcome
Funders Reference	209265/Z/17/Z

1 TRIAL MANAGEMENT TEAM

Trial Administration

Study Coordination Centre: Clinical Trials Facility, Phyles Maitha. Please direct all queries to the SEVUSMART Trial Administrator at KEMRI Wellcome Trust Programme (Phyles Maitha; PMaitha@kemri-wellcome.org) Tel: +254715461761

Clinical Queries

All clinical queries will be passed to Emmanuel Oguda (eoguda@kemri-wellcome.org), Tel: +254748588041 in the first instance at KEMRI Wellcome Trust Programme, who will consult other members of the Clinical Management Group where necessary.

Sponsor

Imperial College London is the main research Sponsor for this study. For further information regarding the sponsorship conditions, please contact the Head of Regulatory Compliance at:

Joint Research Office
Room 221
Medical School Building
St Mary's Campus
Norfolk Place
London W2 1PG

Tel: +44 (0) 020 7594 1872.

Funder

This protocol describes the SEVUSMAART study and provides information about procedures for entering participants. The protocol should not be used as a guide for the treatment of other participants; every care was taken in its drafting, but corrections or amendments may be necessary. These will be circulated to investigators in the study, but centres entering participants for the first time are advised to contact the trial centre to confirm they have the most recent version. Problems relating to this trial should be referred, in the first instance, to the study coordination centre.

This trial will adhere to the principles outlined in the International Conference on Harmonisation Good Clinical Practice (ICH GCP) guidelines. It will be conducted in compliance with the protocol, the Data Protection Act and other regulatory requirements as appropriate.

Coordinating Centre

KEMRI Wellcome Trust Programme	Switchboard:<br>Mobile:	+254 (0) 417 522063/522535<br>+254 (0) 715461761 (Trial manager)
Clinical Trial Facility	Fax:	+254 (0) 417 522390
PO Box 230, Kilifi, Kenya	Email:	SEVU@kemri-wellcome.org

TRIAL MANAGEMENT GROUP

Principal /Chief Investigator:	Prof Kathryn Maitland MBBS PhD	Mobile:<br>Email:	+254733411022<br>kathryn.maitland@gmail.com
Trial Administrator	Phyles Maitha Dip.Business.Mgt	Tel:<br>Mobile:<br>Email:	**+254 417525453 (switchboard)**<br>+254715461761<br>PMaitha@kemri-wellcome.org
Clinical Project Manager:	Emmanuel Oguda Dip.Clin.Med	Tel:<br>Mobile:<br>Email:	**+254 417525453 (switchboard)**<br>+254748588041<br>eoguda@kemri-wellcome.org
Data Manager:	Christabel Mogaka BSc	Tel:<br>Email:	+254 715811661<br>CMogaka@kemri-wellcome.org

KILIFI COUNTY HOSPITAL/KEMRI WELLCOME TRUST RESEARCH PROGRAMME

Local PI	Dr Mainga Hamaluba MBCHB MRCP	Tel:<br>Email:	+254795320422<br>MHamaluba@kemri-wellcome.org
Study Clinician	Dr Nchafatso Obonyo MBCHB	Tel:<br>Email:	+25473322660<br>NObonyo@kemri-wellcome.org

IMPERIAL COLLEGE, LONDON

Principal Investigator:	Prof Kath Maitland MRCP PhD	Tel:<br>Email:	+44 (0) 207 670 4905<br>kmaitland@imperial.ac.uk
Co Investigator	Prof Thomas Williams PhD	Email:	tom.williams@imperial.ac.uk

ZAMBIA			
Tropical Diseases Research (TDR) Centre<br>6th and 7th Floor of Ndola Teaching Hospital building<br>P.O Box 71769,<br>Ndola, Zambia and Nchelenge Hospital, Luapula Province, Zambia			
Principal Investigator:	Dr Mike Chaponda	Tel:<br>Email:	+260 212 620737<br>mikechaponda@yahoo.com
Co-investigator:	Dr Sam Miti	Tel:<br>Email:	+260 212 620737<br>drsammiti@gmail.com
Co-investigator	Dr Luc Kambale Kamavu	Tel:<br>Email:	nespluckam@yahoo.fr

MRC CTU AT UCL

Medical Research Council Clinical Trials Unit at UCL<br>Institute of Clinical Trials & Methodology, 90, High Holborn, 2nd Floor, London WC1V 6LJ			
Principal Investigator:	Prof Diana M Gibb, MD MSc	Tel:<br>Email:	+44 (0) 207 670 4905<br>diana.gibb@ucl.ac.uk
Trial Statistician:	Professor Sarah Walker, PhD MSc	Tel:<br>Email:	+44 (0) 207 670 4726<br>rmjlasw@ucl.ac.uk
Trial Statistician:	Elizabeth George, MSc	Tel:<br>Email:	+44 (0) 207 670 4931<br>elizabeth.george@ucl.ac.uk

2 SUMMARY OF THE TRIAL

Summary Information	Summary Details
**ACRONYM**	SEVUSMART
**Long Title of Trial**	SEVUparin as a potential Adjunctive Treatment in children with severe malaria
**Version**	2.1
**Date**	28th February 2023
**PACTR number:**	**202007890194806**.
**ICREC**	**18IC4513**
**Study Design**	Phase I Safety and Dose Finding Trial: where dose-toxicity will be assessed using the Continual Reassessment Method (CRM) to adapt or inform subsequent doses for each child entering the trial
**Participants to be Studied**	20 children aged 3 months to 12 years hospitalised with malaria (*Plasmodium falciparum* positive malaria slide) plus lactate >2 mmol/L
**Setting**	Kilifi County Hospital/KEMRIWTRP High Dependency Unit and Nchelenge Hospital, Luapula Province, Zambia
**Interventions to be studied**	Sevuparin given as three infusions at 0, 8 and 16 hours after enrolment. Initially two cohorts of 2 participants will receive a dose of 1.5 mg/kg/dose with the plan to escalate to a cohort of 2 participants receiving 3mg/kg/dose and a cohort of 2 participants receiving 6.0 mg/kg/dose. Using the CRM each subsequent patient would then be assigned the largest dose between 1.5 and 6.0 mg/kg/dose (maximum) with an estimated risk of toxicity below the target toxicity level, designated as the maximum tolerated dose (MTD).
**Study Hypotheses**	Sevuparin given in addition to antimalarial treatment, early in the course of admission (<24 hours), blocks merozoite invasion, prevents cytoadherence and transiently de-sequesters infected erythrocytes (which cause microcirculatory impairment) and could thus result in improvements in outcomes from severe malaria for the subgroups at greatest risk and during the period of greatest risk (first day of hospitalisation).
**Primary Outcome Measure**	Activated partial thromboplastin time (APTT)>2.5x upper limit of normal (ULN) (Common Toxicity Criteria grade 3) 1h post any Sevuparin dose
**Secondary Outcome Measure(s)**	Efficacy<br>▪ Change in lactate from 0 to 8 hours<br>▪ Presence of mature infected erythrocytes on the blood films at 8 and 24 hours<br>▪ Parasite clearance time<br>▪ Change in sublingual microcirculation over time<br>Safety<br>▪ APTT 24h post enrolment (absolute level and grade)<br>▪ Development of abnormalities of coagulation indices of grade 2 and above<br>▪ Neurological sequelae through day 28<br>▪ Mortality through day 28<br>▪ Serious adverse events through day 28<br>▪ Grade 3/4 adverse events through day 28
**No of Participants**	20 children
**Duration**	18 months

**Sponsor**	**Imperial College, London**

**Funder**	**Wellcome**
**Trial Manager**	**Emmanuel Oguda**
**Chief Investigator**	**Kathryn Maitland**
**MRC CTU Project Leader**	**Sarah Walker**

2.1 Lay Summary:

SEVUparin in Severe Malaria as a potential Adjunctive Treatment

What is the problem/background?
Even on the best antimalarial treatments (injectable artesunate) many African children with severe malaria have poor outcomes with most deaths occurring early in the course of hospital admission (<24hours). One of the bad things that happen in severe malaria is that red blood cells that are infected with malaria parasites that stick to the very deep parts of our blood vessels. This occurs throughout the body and therefore the blood flow is poor tissues which leads to a build-up of body acids (called lactate). Up to the present there have been no treatments available to prevent or reverse stop red cells sticking to the blood vessels when they have malaria parasites in them.

The SMAART: Severe Malaria in African children: A Research and Trials consortium have identified one key innovation which could improve this. A novel new drug candidate called sevuparin, has been identified and safely tested in adults with malaria in Thailand. Sevuparin acts by preventing malaria parasites getting into red cells in the first place (this means that the malaria parasite cannot survive); it also prevents red cells with infected with malaria parasites from sticking to the blood vessels and also able to 'unstick' red cells with infected with malaria parasites that are already sticking to the blood vessel and causing poor blood flow.

Given its potential to be transformative in improving current outcomes from severe malaria, a large group of specialist doctors in severe malaria research and clinical trials suggested that sevuparin should be tested in children with severe malaria. They have all helped to design this clinical trial, which will be conducted on the high dependency ward in Kilifi, Kenya.

What questions are we trying to answer?
Study design: Phase I Safety and Dose Finding to assess the optimal dose of sevuparin given as a supportive therapy in severe malaria alongside of the usual antimalarial treatments to determine toxic level, adverse events and to identify the highest dose with an acceptable safety profile
Study Population: 20 children hospitalised with severe malaria.

Sample Size: This is a Phase I trial designed to test safety and Dose Finding Trial of sevuparin as a supportive therapy in severe malaria; there is therefore no formal sample size calculation.

Where is the study taking place, how many people does it involve and how are they selected?
The study will be carried out at Kilifi County Hospital, Kenya and Nchelenge Hospital, Luapula Province, Zambia and plans to enroll 20 children. Children enrolled in the study will be aged 3 months to 12 years and presenting to Kilifi County Hospital with severe malaria evidence of malaria (rapid diagnostic test positive or positive malaria slide). The carers of these children will be approached for consent and enrolment in the study. The study drug will be given on the first day of admission only alongside of usual treatments given for children with malaria.

What does the study involve for those who are in it?

- Parental information and consent/parental assent (for emergencies when the child is too sick for full consent).
- Admission to the high dependency ward at Kilifi County Hospital or paediatric ward at Nchelenge
- Hospital, Luapula Province, Zambia
- The sevuparin will be given as 3 infusions on the first day at study enrolment (0 hours), 8 and 16 hours following this. All other treatments for severe malaria will follow the usual guidelines

- Two groups of 2 participants will receive a dose of 1.5 mg/kg/dose with the plan to escalate to a cohort of 2 participants receiving 3mg/kg/dose and a cohort of 2 participants receiving 6.0 mg/kg/dose. The model will then inform each subsequent patient what dose they would then be assigned (between 1.5 and the maximum dose of 6.0 mg/kg/dose)
- All children will have regular (4 hourly clinical and vital sign) monitoring during the period of admission to the high dependency ward and twice daily thereafter until discharge and will be followed up to day 28. Routine blood tests will be done at admission, 8 and 24 hours and at follow up. Additional samples will be taken to check clotting at 0, 1-hour post sevuparin infusion (i.e., 1, 9 and 17 hours) and lactate (the body acid that builds up in severe malaria) will be check regularly at admission, 4, 8 16 and 24 hours.
- The microcirculation, using a small soft probe placed on the gum of a children will be checked three times during admission.

What are the benefits and risks/costs of the study for those involved?

Benefits: Close monitoring of all children on the high dependency unit during admission and follow up to day 28 after discharge. Hospital bills for participants older than 5 years will be covered by the study (covering the costs for standard treatment for severe malaria and related complications).

Risks: One potential risk of sevuparin, as it is heparin-like drug is that it increases one of the blood clotting markers. This was seen in the study in Thailand but was short lived (i.e., only happened for a few hours only) and did not cause any side effects. We are carefully monitoring this blood clotting marker, and this will help us make decisions about whether the child receives the next dose and as weare using a continuous reassessment method to monitor toxicity this will inform the dosages for the children enrolled in the trial.

How will the study benefit society?

The study aims to improve the outcome from severe malaria. It will generate robust feasibility and safety data to support a future larger phase II trial with the aim of improving management of a vulnerable population of children and improving outcomes.

When does the study start and finish? Once we have received ethical approval.

Enrolment will be over 12-15 months and study will end after 2 years.

ABBREVIATIONS/GLOSSARY

ACT	Artemisinin combination therapy
AE	Adverse event
APTT	Activated partial thromboplastin time
AT	Antithrombin
BCS	Blantyre Coma Scale
CI	Confidence interval
CM	Cerebral Malaria
CRF	Case Record Form
CRM	Continuous Reassessment Method
CTC	Common Toxicity Criteria
DIC	Disseminated intravascular coagulation
DLT	Dose limiting toxicity
DMC	Data Monitoring Committee
ECG	Electrocardiogram
ED50	Median effective dose (effective dose for 50% of people receiving the drug)
GMP	Good Manufacturing Practice
HDU	High Dependency Unit
HRP2	*P. falciparum* Histidine Rich Protein 2
IV	Intravenous
KCH	Kilifi County Hospital
KEMRI	Kenya Medical Research Institute
KWTRP	Kilifi Wellcome Trust Research Programme
ICREC	Imperial College Research Ethics Committee
MRC CTU	Medical Research Council Clinical Trials Unit
MTD	Maximal tolerated dose
OR	Odds ratio
PI	Principal Investigator
PK	Pharmacokinetic
PKPD	Pharmacokinetic-pharmacodynamic
PPB	Pharmacy and Poisons Board
RDT	Rapid diagnostic test
SAE	Serious adverse event
SDF	Side stream-dark field
SERU	Scientific and Ethics Review Unit
SMAART	Severe Malaria in African children: A Research and Trials consortium
SOP	Standard Operating Procedures
TAT	Thrombin-AT III
TF	Tissue factor
TM	Thrombomodulin
ULN	Upper limit of normal

3 ABSTRACT

3.1 Abstract

Severe Malaria In African Children: A Research and Trials Consortium (SMAART) was funded in 2018 by a collaboration award in science from the Wellcome Trust. Owing the continued high burden of malaria and poor outcomes of severe malaria, even on the best antimalarial, artesunate, the major objective of SMAART is to conduct better research faster across a collaborative platform to identify interventions to optimise the whole treatment pathway for children with severe malaria, and thus to achieve a step-change in improving their outcomes in the current era.

A novel new drug candidate for adjunctive treatment of severe malaria, sevuparin, has been identified that can blocks merozoite invasion, prevent cytoadherence and transiently de-sequesters infected erythrocytes in humans with uncomplicated *P. falciparum* malaria. If given, in addition to antimalarial treatment, early in the course of admission (<24 hours) this could result in improvements in outcomes from severe malaria for the subgroups at greatest risk and during the period of greatest risk (first day of hospitalisation). Sevuparin has been shown to be safe and well tolerated in adults with only some mild effects on activated partial thromboplastin time (APTT) at higher doses given over longer periods of time (3 days), which are not clinically relevant to the time of greatest risk.

In this Phase I trial dose-finding paediatric study, we aim to use only 3 doses given at admission (0 hours), and 8 and 16 hours later, and measure APTT 1 hour after each dose (to assess maximum toxicity based on adult data). The Phase I trial is designed to obtain data on safety, dosing, feasibility and potentially lactate clearance of sevuparin given as an adjuvant therapy in severe malaria in children. We aim to study 20 children admitted to hospital with severe malaria which will allow sufficient data on safety to be generated across a range of doses to identify the maximum tolerated dose (MTD) using the Continual Reassessment Method (CRM), which adapts or informs subsequent doses for each child entering the trial based on the data from previously enrolled children. The maximum tolerated dose (MTD) will be identified based on the dose-toxicity model being updated by each previous patient's APTT results using standard methods.

The results of the Phase I trial will identify the final dosage to be tested in a Phase II trial in terms of both efficacy and safety outcomes. The primary endpoint for the future Phase II trial (lactate clearance) reflects the primary hypothesis that sevuparin improves microcirculatory flow by reversing and preventing parasite sequestration. The Phase I trial will also assess whether this endpoint, lactate clearance, is realistic; thus, lactate will be collected along with other test results at baseline 4 hours and 8 hours, as well as other time points.

4 TABLE OF CONTENTS

2.1	Lay Summary:	7
3.1	Abstract	10
5.1	Background	13
5.2	Treatment of severe malaria	13
5.3	Severe Malaria in African children A Research and Trials Consortium (SMAART)	13
5.4	Pathophysiology of severe malaria and metabolic acidosis	14
5.5	Adjuvant or supporting treatments aiming at improving the microcirculation	15
5.6	What is known about coagulation in children with severe malaria	17
7.1	Hypothesis	20
7.2	General Objectives	20
7.3	Specific Objectives	20
8.1	Inclusion criteria	21
8.2	Exclusion criteria	21
9.1	Sample Size determination	22
9.2	Sampling Procedures	22
9.3	Study Procedures	23
9.4	Consent process	23
9.5	Trial Treatments	23
9.6	Clinical Endpoints	25
9.7	Interim reviews	26
11.2	Reporting Procedures	31
12.1	Data Storage	33
12.2	Data Management and Statistical Analysis	33
12.3	Intellectual Property	33
12.4	Time Frame/Duration of the Project:	33
13.1	Human subjects	34
13.2	Patient Consent	35

**13.3**	**WITHDRAWAL OF PATIENTS and Protocol Treatment Discontinuation**	**35**
**13.4**	**Protocol Treatment Discontinuation**	**36**
**13.5**	**Community Engagement Strategy and feedback**	**36**
**13.6**	**Compensation**	**36**
**13.7**	**Confidentiality**	**37**
**13.8**	**Data Sharing**	**37**
**13.9**	**Indemnity**	**37**
**13.10**	**Sponsor**	**37**

5 INTRODUCTION/BACKGROUND

5.1 Background

Over the last decade there has been an unprecedented rise in funding for malaria control activities, including the scale-up in long-lasting insecticidal bed nets and introduction and access to effective artemisinin-combination treatments (ACT). This has resulted in the disease retreating from large parts of the globe, yet malaria remains stubbornly unyielding in sub-Saharan Africa and in some parts of Asia [1]. In some African countries, e.g., Nigeria, DRC, Uganda, Mozambique, the pattern of transmission has not changed appreciably, despite implementation of early treatment and control strategies and they continue to contribute most to the global disease burden [2]. Many of the African countries most afflicted by malaria are amongst the poorest on the continent with weak health services infrastructure. Severe malaria remains a major public health problem in Africa and a chief factor in child mortality; particularly in countries experiencing high transmission. In 2016 it was estimated that there were over 445,000 deaths from malaria with the vast majority of these deaths in African children [3, 4]. Whilst the AQUAMAT trial has provided definitive evidence for optimal antimalarial treatment in severe malaria [5], progress on improving outcome through supportive treatments has been very slow [6, 7].

5.2 Treatment of severe malaria

The multi-centre AQUAMAT trial conducted in 11 centres in 9 countries in Africa compared quinine and artesunate in 5425 children hospitalised with severe malaria. The primary endpoint, in-hospital mortality, in the intention to treat analysis occurred in 297/2713 (10.9%) children receiving quinine treatment compared to 230/2712 (8.5%) children receiving artesunate - translating to a relative reduction in mortality of 22.5% (95% CI 8.1-36.9) with artesunate (p=0.002) [5]. However, outside the context of a clinical trial, overall in-patient mortality for severe malaria remains unacceptably high (~10%), and unlikely to improve without wider implementation of pre-referral artemisinin [8] and better supportive treatments [6, 9]. As demonstrated in Table 1 below, case fatalities in AQUAMAT were even higher within large subgroups of patients presenting with one or more of the 3 key prognostic markers (coma, acidosis, or a high blood urea nitrogen [10]). The evidence base to guide best management of these and other complications is lacking. To date none of 33 clinical trials of adjunctive (supportive) treatments conducted globally since a seminal severe malaria trial in 1980 have shown benefit [6]. Over 60% of these trials involved children and 15 were specifically directed to the sub-group with cerebral malaria. The majority were single-centre Phase I or II trials involving few participants, and a reasonable number were stopped prematurely because they showed harm.

5.3 Severe Malaria in African children A Research and Trials Consortium (SMAART)

Severe Malaria In African Children: A Research and Trials Consortium (SMAART) was funded in 2018 by a collaboration award in science from the Wellcome Trust. The major objective of the collaborative award was to identify which interventions could optimise the whole treatment pathway for children with severe malaria from the hospital ‘gateway’ to survival 6-months post-discharge, and hence achieve a step-change in improving their outcomes in the current era. A 2-day inception meeting of the SMAART consortium brought together a multidisciplinary group of scientists encompassing

decades of clinical and academic experience, with track records of successful high-quality research in low-income settings in severe malaria. The group considered the high priority areas of research and key targets for intervention. One high risk group identified by the group was children presenting with acidosis (increased base excess); in this sub-group mortality remained at 15%, and acidosis was one of the three key factors predicting poor outcome [10].

Table 1 High priority risk factors for severe malaria and recent trials

Admission feature or complication	Frequency	AQUAMAT in-hospital Mortality* (Artesunate-arm)	
Coma	32-35%	18%	
Metabolic acidosis (base excess<-8 or lactate>5mmol/L)	43-44%	15%	
Renal impairment (Urea/BUN > 20 mmol/L)	24%	22%	
Hypoglycaemia (blood glucose <3 mmol/L)	10%	15% (DOI 10.1186/1471-2334-10-334)	
Convulsions	30-32%	14%	
Invasive bacterial co-infection	5.5%	24% (DOI: 10.1186/1741-7015-12-31)	
Blackwater Fever (region specific)	14-21%	Day-28 mortality 12% (DOI:10.1093/cid/cix003)	
**Recent or ongoing trials**	**Frequency**	**Mortality**	**Trial:** results expected
Shock (mortality = no-bolus arm)	12%	8.5%	**FEAST:** 10.1056/NEJMoa1101549
Severe anaemia	29-30%	10%	**TRACT:** July 2018 ISRCTN84086586
Hypoxaemia (<90%)	15%-17%	14%-30%	**COAST:** Late 2020 ISRCTN15622505

* Data from AQUAMAT unless indicated where mortality figures are for quinine-treated children

In order to identify supportive therapies which could target acidosis, the following section summarises the current understanding of the pathophysiology of severe malaria and likely aetiology of 'malaria' acidosis.

5.4 Pathophysiology of severe malaria and metabolic acidosis

During the course of infection, ring stage parasitaemia or infected erythrocytes in children with *P. falciparum* is amplified. Unlike other malarias species, *Plasmodium falciparum* has the unique ability to cause cytoadherence, a phenomenon called sequestration, of late stage parasitised infected erythrocytes in the deep vascular beds [11]. The pathophysiological process is mediated by excessive sequestration of *P. falciparum* infected erythrocytes [12], rosetting [13] and decreased deformability of non-parasitised red cells [14]. Whilst this can occur during a non-severe infection, autopsy studies have shown that there is intense sequestration of parasitized erythrocytes in vital organs in children who have died from malaria (i.e., had severe malaria), but sequestration varies between organs, and even varies even within an organ, with some vessels completely blocked while other proximate vessels remain patent [14, 15]. Whether sequestration causes mechanical obstruction and impaired tissue perfusion or is damaging in other ways (active parasite metabolism, release of toxins, cytokine induction) [16] is not known.

One marker associated with poor outcome is a raised lactate, which is generally considered to be directly linked to the degree of impaired perfusion [17]. Evidence to support this include the fact that improvements in lactate concentration over the first 24 hours of admission were strongly prognostic for survival in adults with severe malaria [18]. Moreover, faster clearance of plasma lactate was predictive of the treatment effect on mortality of artemether compared to quinine [19]. Artemether results in a rapid killing of ring stage parasites, preventing their further maturation and sequestration in the microcirculation and this is thought to be a main contributor to the improvement in case fatality. In the fluid resuscitation (FEAST) trial that included a large number of children with severe malaria and sepsis, a sub-analysis showed that severe lactataemia (>5 mmol/L) was strongly associated with mortality (Odds Ratio (OR) 6.96; 95% CI 3.52, 13.76, p<0.001) and that failure to clear lactate at 8 hours was strongly associated with death at 72h (OR 4.62; 95% CI 2.7, 8.0; p < 0.001) [20].

5.5 Adjuvant or supporting treatments aiming at improving the microcirculation

The demonstration in autopsy studies of microvascular obstruction by heavy parasites burdens [21] and that an overall measure of parasite biomass (*Plasmodium falciparum* histidine-rich protein2 (pfHRP2), a protein released on sequestration by infected erythrocytes) correlates with worse outcomes [22] suggest that adjuvant therapies which can reverse sequestration and reduce overall biomass (by preventing merozoite invasion) early in the course of the disease may lead to substantial improvements when the risk of fatal outcome is highest. Moreover, the time of development of a merozoite into an adhesive infected erythrocyte that sequesters and blocks the micro-vasculature is ~18-20 hours which is the same time period (first day of hospitalization) when the majority of paediatric deaths from severe malaria in Africa occur.

In studies conducted in Indonesia, it was hypothesized that heparin could inhibit *P. falciparum* sequestration and merozoite invasion since heparin binds to heparan sulphate binding structure of *Plasmodium falciparum* erythrocyte membrane protein 1 (PfEMP1), the Duffy-binding like domain 1α (DBL1α), known as a vital contributor to sequestration of infected erythrocytes [23, 24]. Clinical trials showed that heparin, as an adjunctive therapy to antimalarial drugs, had variable results, with some showing reduced mortality in children with severe *P. falciparum* malaria [25, 26], others showing no clinical improvements when given as a low dose [27] and others demonstrating potentially severe effects on coagulations in simian studies in *P. knowlesei* malaria [28, 29] at the same dosage as those showing benefit in children with severe malaria. As a result, heparin was not subsequently adopted into clinical practice owing to the substantial concern over haemostatic side effects. Subsequent investigation has shown that that the inhibitory effect of heparin on *P. falciparum* sequestration and merozoite invasion (which also is mediated through the heparan sulphate binding site of PfEMP) is independent of its anti-coagulant activity [30, 31]. The next step was to develop a heparin compound that was devoid of its therapeutic limiting effects on coagulation.

5.5.1 Sevuparin

The drug sevuparin was developed from heparin because its heparan sulfate binding is nearly identical; thus, the rationale was that sevuparin would act as a decoy receptor during malaria infection [32]. Sevuparin, akin to other heparins, is a poly-disperse chemical, encompassing a range of polysaccharide chain lengths with molecular weights of 3.6–9.6 kDa. Sevuparin is negatively charged and derived from

heparin through chemical depolymerization. In sevuparin, the specific pentasaccharide involved in high-affinity binding to antithrombin III has been deleted. Thus, since sevuparin has no specific binding sequence for antithrombin (AT) which is the main contributor to prolonged coagulation, it has no direct effect on factor Xa or on thrombin, and its effect on activated partial thromboplastin time (APTT) is markedly reduced compared to that of standard dose heparin [33]. For example, for it to have the same effect (ED50) on APTT prolongation (measured as ED50), sevuparin would need to be given in doses five times higher compared to low molecular weight heparin and 35 times higher compared to full length heparin.

Like heparin, sevuparin binds to the heparin sulphate binding structure of Plasmodium falciparum erythrocyte membrane protein 1 (PfEMP1), which is the key receptor for sequestration of infected erythrocytes [34, 35]. Preclinical investigations have demonstrated that sevuparin blocks *P. falciparum* merozoite invasion into fresh erythrocytes *in vitro*, and both disrupts and blocks the binding of infected erythrocytes to uninfected erythrocytes (rosetting) and binding to vascular endothelial cells (also known as cytoadherence) *in vitro*. *In vivo* studies also demonstrated that sevuparin led to de-sequestering of schizonts both rats and in non-human primate preclinical studies [25-27] and to disruption of rosetting in a dose-dependent manner [34].

Phase I studies in healthy volunteers and a clinical study of adults with sevuparin have been conducted in patients with mild *P. falciparum* malaria have been conducted in which it was proposed that sevuparin would act as a decoy receptor during malaria infection to block merozoite invasion as an adjunctive therapeutic approach to preclude the early expansion of an infection and reduce the sequestered biomass [36].

In the Phase I dose escalation trial, sevuparin was found to be safe and well-tolerated with minimal effects on APTT at doses of 1.5 mg/kg and 3mg/kg every six hours but a dose of 6.0 mg/kg led to higher APTT values. The Data Monitoring Committee (DMC) therefore recommended that the study should continue to the Phase II with a dose of 3.0 mg/kg sevuparin every 6h, given the potentially increased risk of adverse events at higher doses [36].

In the Phase II trial, sevuparin was administered as an intravenous infusion over 5 minutes in addition to standard care i.e., a fixed dose combination of 1000 mg atovaquone and 400 mg proguanil once daily for three days, in subjects with uncomplicated *P. falciparum* malaria (experimental arm), as compared to atovaquone/proguanil treatment alone (control). There were minimal and non-clinically relevant changes in anti-Xa- and prothrombin-times, and the international normalized ratios associated to the sevuparin dose [36] (see Supplementary Tables S1 and S2 from the manuscript). Increases in APTT were dose-dependent and appeared to follow the time-concentration curve for each sevuparin infusion. The 6-hour interval between the infusions allowed for nearly full reversibility of APTT levels after each dose, and no accumulative effects were seen over the course of the 12 consecutive infusions (see Supplementary Tables S3 and S4 from the manuscript) [36]. Thrombocytopenia occurred in one subject but was noted to be present before the initiation of sevuparin treatment. There was no incident of bleeding in any of the participants nor were there any other serious adverse events or adverse events.

Sevuparin was therefore judged to be safe and well tolerated in adults with mild malaria. It led to a reduction in numbers of ring-stage infected erythrocytes after a single sevuparin infusion and resulted

in a transient appearance of mature parasite infected erythrocytes (schizonts appearing in the circulation, indicating these had been released after desequestration). These both occurred within one hour after the first sevuparin injection. Thus, these studies indicate that a novel new drug candidate for adjunctive treatment of severe malaria had been identified that blocks merozoite invasion and transiently de-sequesters infected erythrocytes in humans with uncomplicated *P. falciparum* malaria.

5.6 What is known about coagulation in children with severe malaria?

5.6.1.A General Background

Normal haemostasis is a complex balance between pro- and anti-thrombotic pathways [37]. Most reactions take place on endothelial or platelet cell surfaces and involve soluble coagulation factors and natural inhibitors. The 'intrinsic' and 'extrinsic' pathways of the coagulation cascade are no longer thought of as separate processes that occur in the plasma. Instead, these reactions occur in concert on membrane surfaces provided by endothelial cells, platelets, monocytes and neutrophils [38]. The recruitment and tethering of these cellular components is a vital step in the initiation of the procoagulant pathways. Endothelial injury, whether due to trauma, inflammation or infection causes activation of three main pro-coagulant pathways: the coagulation cascade, platelet reactions and vasoconstriction. In severe *P. falciparum* malaria, adhesion molecule upregulation has been demonstrated [39, 40] and thrombomodulin levels (TM) have been reported to be high [40, 41] suggesting that any coagulation activation seen might be due to endothelial dysfunction. Few detailed studies exist of coagulation abnormalities in severe malaria; frank disseminated intravascular coagulation (DIC) is rare despite thrombocytopenia being common [42-44]. Early studies of the mechanisms involved in the activation of the coagulation cascade in severe *falciparum* malaria in Thai adults, many of whom had multiple vital organ dysfunction, suggested activation of the intrinsic pathway of the clotting cascade and complement system including reduction in the concentration of plasma antithrombin III (AT III) concentrations, elevation in thrombin-AT III (TAT) complexes [45, 46], and reductions in factor XII and prekallikrein activities. Protein-C activity was also shown to be reduced [46]. Subsequent studies have also shown that *P. falciparum* malaria is associated with procoagulant activity but not with clinical evidence of thromboembolism. Plasma levels of TM have been used to assess the participation of the vascular endothelium in human *falciparum* malaria. Studies in adults have shown that elevated plasma levels of TM correlate directly with the levels of parasitaemia, TNF alpha, elastase and TAT [41, 47]; and that the low plasma levels of Protein-C and protein-C inhibitor-1 and increased TAT concentrations present in almost all patients correlated with severity and parasitaemia [41, 47]. Together these data suggest that there is endothelial activation and a shift towards a pro-coagulant state in *P. falciparum* malaria, both of which can be reversed after anti-malarial treatment [47].

5.6.1.B Paediatric Studies

Studies of coagulation in African children with severe malaria are relatively few but clinical evidence of DIC is rare [48]. In paediatric cerebral malaria (CM), autopsy studies have shown that fibrin degradation products are raised [49], indicating a pro-coagulant state. These studies also showed a consistent staining for tissue factor (TF) in the endothelial cells and TF was also shown to be

upregulated in the brain postmortem studies in paediatric CM [50]. However, the latter study also showed that TF was also found in postmortem samples from parasitaemic children whose underlying illness was non-malarial [50]. Moreover, when comparing functional coagulation assays in African children with CM to children with mild malaria, no marked differences were found [51].

A more recent and comprehensive case-control study of coagulation compared a range of indices in children with true cerebral malaria (defined by a malarial retinopathy) compared with children with other forms of severe malaria, mild malaria and healthy controls. Compared to healthy controls (n=19), TAT, a sensitive marker of thrombin generation, was increased in children with retinopathy-positive CM (n=66) ($P<0.001$) and levels were greater than those in children with uncomplicated malaria (n=30) ($P<0.01$). In the retinopathy-positive CM group, TAT levels were higher in 16 fatalities than in those children who survived. Prothrombin times were mildly and similarly prolonged in both CM children and children with uncomplicated malaria compared to healthy controls [52]. APTT levels were similar to the controls in all malaria groups [52], indicating activation of coagulation through TF activation rather than increased factor XII [46].

6 RATIONALE

A novel new drug candidate for adjunctive treatment of severe malaria, sevuparin, has been identified that can block merozoite invasion, prevent cytoadherence and transiently de-sequester infected erythrocytes in adults with uncomplicated *P. falciparum* malaria. If given, in addition to antimalarial treatment, early in the course of admission (<24 hours) this could result in improvements in the outcome from severe malaria for the subgroups at greatest risk and during the period of greatest risk (first day of hospitalisation). Sevuparin has been shown to be safe and well tolerated with only some mild effects on APTT levels at higher doses given over a longer period of time (3 days), which is not clinically relevant to the time period of greatest risk (first day of hospitalization). In this Phase I trial dose-finding paediatric study, we aim to use only 3 doses given at admission (0 hours), and then 8 and 16 hours subsequently, and will measure the key toxicity of interest, APTT, 1 hour after each dose (to assess maximum toxicity). The normal ranges of APTT in children have been shown to be the same those in as adults [53] so this study will learn from and build upon what has already been published on sevuparin in adults with malaria. A large comprehensive study of coagulation abnormalities in African children with severe malaria, mild malaria and healthy controls demonstrated that there are no derangements in APTT in children with severe malaria compared to mild malaria and healthy controls [52], providing reassurance that the comparison of APTT levels with normal ranges in this study is clinically meaningful in terms of identifying a maximal tolerated dose (MTD). Finally, if sevuparin resulted, in a future Phase III trial, in increased disability-free survival, then this may be a future suitable candidate to use in pre-referral management alongside parenteral or artesunate.

The design of this dose finding study uses the Continual Reassessment Method (CRM). This adaptive dose-finding study design is increasingly embraced by clinical trialists [54] as a more efficient method for identifying an “optimal” dose using as small a number of participants as possible, in contrast with heuristic methods such as, for example, comparing three arbitrarily chosen doses. The CRM ‘learns’ (i.e., reassesses risk/toxicity) after each patient is entered into the trial and proposes a subsequent dose for the next child entered in a way that provides the most information about doses closest to the

MTD. The CRM and has been shown to incur fewer toxicity events overall in identifying the MTD, and to estimate the MTD more accurately as compared the standard Phase I dose escalation designs [55]. In terms of safety, the study will be run using a 'live' Data Monitoring Committee (DMC) review of toxicity events. The DMC will review data after the first 2 'cohorts' of 2 children each have been enrolled to the lowest dose (1.5 mg/kg/dose), and then after each Dose Limiting Toxicity (DLT) event.

7 TRIAL DESIGN

7.1 Null Hypothesis

We hypothesize that sevuparin, a de-polymerised heparan sulphate mimetic, will not improve microcirculatory flow by reversing and preventing parasite sequestration when given to children with severe malaria and will not improve overall outcome.

7.2 General Objectives

The primary objective of this trial is to conduct a dose-finding study of intravenous sevuparin given in 3 doses over the first 18 hours from enrolment (within 24h of hospital admission), defining toxicity events as any APTT >2.5 upper limit of normal (ULN) (grade 3 toxicity) 1 hour after each dose to identify the maximum tolerated dose (MTD). The initial dosage (1.5 mg/kg) and the *a priori* dose-toxicity curve are based upon the results of the adult trial (where a dose of 1.5 mg/kg was associated with minimal risk of toxicity) and experimental evidence of dose-dependent efficacy i.e. inhibition of merozoite invasion and reversal of cytoadherence of infected erythrocytes [36]. Almost all adults enrolled in this trial experienced grade 2 toxicity after one or more sevuparin doses, but APTT rapidly normalized, hence the choice of grade 3 toxicity to define the MTD in this dose-finding trial.

The results of this Phase I trial will identify the final dosage selected for a subsequent Phase II that will include both efficacy and safety outcomes. The Phase II trial will be conducted by the SMAART consortium and plans to use change in lactate at 8 hours as its primary endpoint; likely secondary endpoints include neurological sequelae, day-28 and day-180 mortality, length of initial hospitalisation, re-admission to hospital, grade 3/4 and serious adverse events.

The primary endpoint for the future Phase II trial reflects the primary hypothesis that Sevuparin improves microcirculatory flow by reversing and preventing parasite sequestration. Data collected in the Phase I trial will also assess whether lactate clearance at 8 hours is a realistic and feasible primary endpoint for a subsequent Phase II trial.

7.3 Specific Objectives

To identify the maximum tolerated dose (MTD) of intravenous sevuparin as an adjunctive therapy in children with severe malaria given as three infusions at 0, 8 and 16 hours using the Continual Reassessment Method (CRM) to adapt or inform subsequent doses for each child entering the trial, based on a toxicity event defined as any APTT >2.5 upper limit of normal (ULN) 1 hour after each dose, and updating the dose-toxicity model using the previous patients' APTT results.

The secondary objective will be to assess whether lactate clearance at 8 hours is a realistic and feasible primary endpoint for a subsequent Phase II trial.

8 LOCATION AND STUDY DESIGN

Site: High dependency ward in Kilifi County Hospital, Kenya or the paediatric ward at Nchelenge Hospital, Luapula Province, Zambia

Study design: Phase I trial

Study population: 20 children hospitalised with severe malaria

8.1 Inclusion criteria

1. Aged between 3 months and 12 years admitted to the paediatric wards within the last 24h
2. Current evidence of *P. falciparum* malaria (slide positive)
3. Clinical evidence of severe malaria: impaired consciousness: coma (inability to localize painful stimulus) or prostration (inability to sit unsupported for those above 6 months) or deep breathing
4. Lactate > 2 mmol/L
5. Guardian or parent willing and able to provide consent

8.2 Exclusion criteria

1. Clinical evidence or a history of a bleeding/coagulation disorder
2. A comorbidity which clinician believes has a significant risk of poor outcome e.g., malignancy,end-stage renal failure, major cardiac condition
3. Thrombocytopenia (platelet count <25 x10^9/L).

9 PROCEDURES

9.1 Sample Size determination

This is a Phase I trial designed to obtain data on safety, dosing, feasibility, and lactate clearance of sevuparin given as an adjuvant therapy in severe malaria. We aim to study 20 children since this will allow sufficient data on safety to be generated across a range of doses to identify the maximum tolerated dose (MTD) from a more informed model relating dose to toxicity events (denoted the 'dose-toxicity' curve) than that available *a priori* based on published data from adult studies. After each patient is enrolled, the dose-toxicity curve will be updated based on levels of APTT taken over three time points (1h post each infusion), defining a toxicity event as APTT >2.5xULN at any time point (grade 3 following the Common Toxicity Criteria (CTC)). This enables the MTD to be estimated more rapidly using the Continuous Reassessment Method (CRM): once determined, subsequent participants will be allocated to this MTD to provide the most accurate estimate of future toxicity event rates until we reach the sample size of 20 children. However, the CRM method will continue to use information from all these children; for example, if a number of children receiving the originally identified MTD experience toxicity events, the dose would again be lowered, and future children would receive this lower dose.

9.2 Sampling Procedures

Eligible children will have been admitted to the High Dependency Unit in Kilifi so children can be closely monitored. Once eligibility is verified, the parents can be approached for consent and, if they agree to participate, they will receive their allocated treatment dosage.

The blood tests taken at admission and during the trial include standard of care and research bloods (see Table 2). Admission and serial assessment of full blood count, admission point of care clinical chemistry including PH, blood cultures and repeated assessment of malaria parasitaemia are part of the standard clinical tests. Additional to this will be serial assessment of lactate, measurements of coagulation (by iSTAT *(*Kaolin ACT) and laboratory based APTT) and samples will be stored in Kilifi for future pharmacokinetic (PK) tests, plasma HRP2 tests and malaria parasites(research). The reliability of Point of care ACT measurements (including ISAT) have previously assessed against gold standard and have shown that the coefficients of variation of POC PT and whole blood were between 2% and 3.6%, indicating that POC assessments are reliable and able to support on-site decision-making for patients in acute and intensive care [56].

Management and outcome data will be collected (clinical parameters and recovery, developmental assessment, number of transfusions, use of drugs (specifically anticonvulsants, paracetamol, and antibiotics), date of discharge or in-hospital death. Contact and locator data will be recorded so that children can be followed at day 7 and day 28.

9.3 Study Procedures

All children admitted with suspected malaria and either coma or respiratory distress (defining severe malaria for this Phase I study) will be screened for study inclusion by the paediatric triage/admission team.

9.4 Consent process

Once eligibility has been confirmed, authorized trial staff will approach parents/guardians to invite their child to take part in the trial. An information sheet will be provided to the parent/guardian in their usual language. The sheet will be read aloud to those who are unable to read. The doctor/nurse will check that the information has been fully understood and parents/guardians will be encouraged to ask questions they may have about their child's participation.

Where possible, prospective written informed consent will be sought from parents/guardians by asking them to sign the Consent Form. If parents/guardians are unable to sign, a thumbprint will be taken in lieu of a signature. A copy of the Consent Form will be given to the parent/guardian, the original placed in the patient's medical notes, and a copy kept in the Investigator Site File.

If it is considered that the full consent process would significantly delay enrolment, and consequently be detrimental to the child's health, then emergency verbal assent, used in the FEAST, TRACT and COAST trials [57-59], will be sought from parents/guardians by the admitting medical team. Following verbal assent, written informed consent will be sought from the parent as soon as possible once the child's clinical condition has stabilized.

9.5 Trial Treatments

We aim to study 20 children. All children will receive standard care including parenteral artesunate and sevuparin given as three infusions at 0, 8 and 16 hours after enrolment. The initial participants (two 'cohorts' of 2 children each, i.e., 4 children in total) will receive a dose of 1.5 mg/kg/dose with theplan to escalate up to a cohort of 2 children receiving 3mg/kg/dose and a cohort of two children receiving 6.0 mg/kg/dose (maximum). In order to determine whether, and the rate of escalation, to a higher dosage for subsequent patients after the first 4 children we will use a design called the Continuous Reassessment Method (CRM). This starts with an estimated dose-toxicity curve, reflecting the probability of experiencing a toxicity event (here APTT>2.5xULN 1h after any of the three doses) to the dose received. In this method of dose finding, the dose-toxicity curve is fitted to the data after each child is enrolled, based on their observed dose and toxicity outcome. After the first 8 children, each subsequent patient would be assigned the largest dose with an estimated risk of toxicity below the target toxicity level, designated as the maximum tolerated dose (MTD). The dose limiting toxicity (grade 3 APTT, >2.5xULN) *a priori* dose-toxicity curve and chosen target toxicity rate (15%) used in this trial have been based upon data shared by the investigators from the adult sevuparin studies in Thailand [36]. In particular, almost all adults enrolled in this trial experienced grade 2 toxicity after one or more sevuparin doses, but APTT rapidly normalized, hence the choice of grade 3 toxicity to define the MTD in this dose-finding trial. APTT 24h post-enrolment is a key secondary safety endpoint to confirm rapid normalization.

Any child with APTT>2.5xULN 1h after a dose of sevuparin will immediately discontinue trial treatment (and be counted as a toxicity event). They will continue to be followed according to the trial schedule (on-study, off-study-drug), to confirm resolution of APTT and to record clinical outcomes.

9.5.1. A Sevuparin

The drug product, sevuparin 150 mg/mL solution for intravenous (IV) infusion, is formulated in a 0.015M phosphate buffer at a pH of 7.0. It requires storage in a refrigerator at 2-8° and to be protectedfrom light. The non-preserved sterile solution needs to be dispensed (5.4 mL) in a glass vial sealed witha rubber stopper and covered with a tear-off aluminum cap. The solution for administration will be prepared in a syringe and will be kept refrigerated and used within 24 hours. One vial will only be usedfor one subject.

The drug product is produced in compliance with current Good Manufacturing Practice (GMP). The same material and compositions are being used by the Sponsor in a currently ongoing clinical trial in Sickle Cell Disease (see below). We will obtain the product from MODUS, Sweden.

Sevuparin is currently in clinical phase II for treatment of acute painful Vaso-Occlusive Crisis in hospitalized Sickle Cell Disease patients (TVOC01 study - clinicaltrials.gov/ct2/show/NCT02515838). This study is expected to complete enrolment in 2018. Children 12 – 16 years of age are included in this study (as are adults), but younger children are not eligible. It is not clear what dose is being used in eligible adolescents (or adults).

9.5.2 Assessments of microcirculation

The sublingual microcirculation will be assessed at the Kilifi site only using sidestream-dark field (SDF) imaging (CytoCam, Braedius Medical BV®). SDF imaging technology is a validated real-time visualization of the microcirculation using a 2ms pulsed green light emitted at a 530nm wavelength for optimal optical absorption by the haemoglobin in red blood cells, independent of oxygenation

state. It is safe, non- invasive and can be performed rapidly at the patients' bedside.

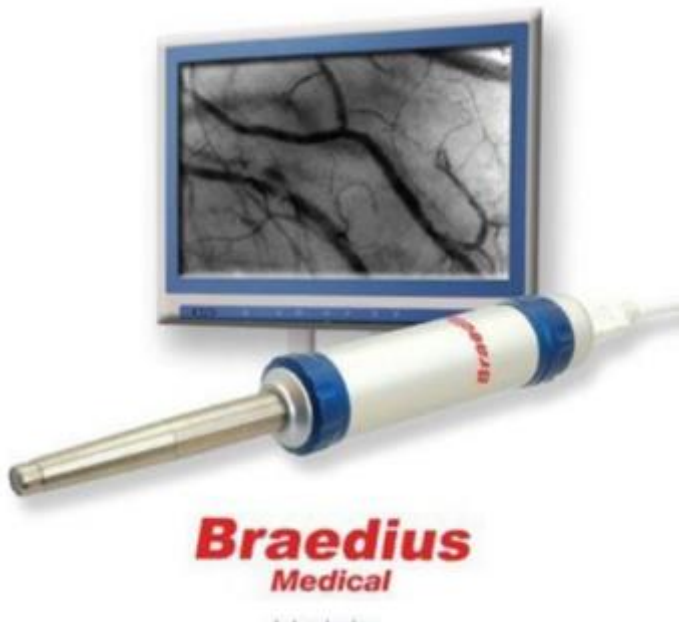

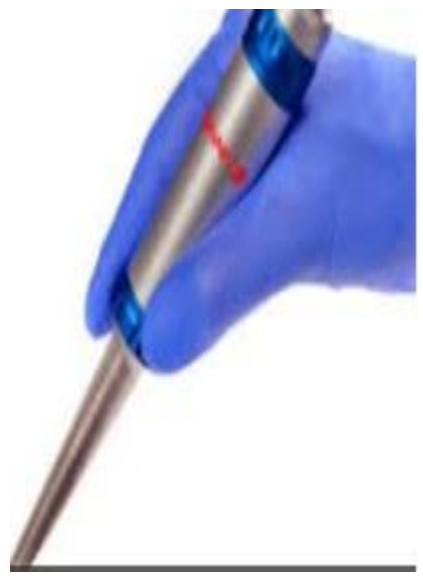

A sterilised disposable lens is used to prevent contact between the instrument and patient and therefore to prevent any transmission of infection. Two trained individuals will collect this data to limit inter-user variability. Images will be recorded from the proximal, mid, and distal portions in each half of the sublingual mucosa and averaged. Where possible the microcirculation will be assessed at time 0 (prior to sevuparin dose), at 8-9 hours (before and after infusion) and again at 17-18 hours (after final dose). The image acquisition and analysis used for assessment of the microcirculation will be in line with the 2007 consensus agreement. This will include a Capillary Network Analysis for vessel densities (total and perfused), the proportion of perfused vessel and the average perfusion speed indicator.

9.5.3 Assessments of Pharmakokinetics of Sevuparin

An aliquot of plasma from the samples taken during the trial will allow pharmacokinetic-pharmacodynamic (PKPD) modelling of the relationships between drug levels and longitudinal APTT and plasma lactate levels. The evaluation of the PK data will focus on the association between the sparsely sampled sevuparin concentrations and the APTT levels, plasma lactate levels and renal function. These assays will be contracted to Accelera (Nerviano (MI), Italy) since they have validated methodology for Sevupain measurement and the data sent to MORU, Bangkok who will undertake tbe PK modelling at the end of the clinical study.

9.6 Clinical Endpoints

9.6.1 Primary Endpoint

- APTT>2.5xULN 1h post any sevuparin dose (grade 3 following the CTC)

9.6.2 Secondary Endpoints

9.6.2.A Efficacy

- Change in lactate from 0 to 8 hours
- Presence of mature infected erythrocytes on the blood films at 8 and 24 hours
- Parasite clearance time
- Change in sublingual microcirculation over time

9.6.2.B Safety Endpoints

- APTT 24h post enrolment (absolute level and grade)
- Development of abnormalities of coagulation indices (prothrombin) (Grade 2 and above)
- Neurological sequelae through day 28

- Mortality through day 28
- Serious adverse events through day 28
- Grade 3/4 adverse events through day 28

APTT 24h post enrolment will be used as an assessment of normalization 8h after the final sevuparin dose. Both absolute levels and grade will be considered.

De-novo evidence of neurological sequelae will be ascertained using a modified Kilifi Developmental Index [60] assessed at admission (to identify pre-existing conditions) and follow up (which we have adapted to use for the COAST trial) [59].

Serious adverse events will use the standardized definitions (see section 11 below). SAEs will be independently reviewed in real-time by the DMC.

Adverse events will be graded following the Common Toxicity Criteria v5.0 (https://ctep.cancer.gov/protocoldevelopment/electronic_applications/docs/CTCAE_v5_Quick_Reference_5x7.pdf).

9.7 Interim reviews

The DMC will review data after the first 2 'cohorts' of 2 children each have been enrolled to the lowest dose, and then after each Dose Limiting Toxicity event. Professor Timothy Peto (Oxford) has agreed to be the Chairman is the DMC, other members will include an independent statistician and 2 pediatricians with relevant DMC or clinical trial experience. They will meet by regular teleconference. The continual reassessment method uses data from each enrolled participant to update the dose-toxicity curve and then suggests an escalation of dose to the largest dose with risk of estimated risk of toxicity below the target toxicity level if appropriate.

10 CLINICAL MANAGEMENT

Members of the clinical team and study team will all receive pre- and peri-trial training on the management of severe malaria. A manual of operations for the trial will be available for the study team, to anticipate and troubleshoot any potential issues.

Children will initially receive parenteral antimalarial treatment (artesunate), followed by on day 3 (or when the child can safely take and retain oral feeds and fluids) an oral course of artemisinin combination therapy (ACT). All trial patients will receive intravenous antibiotics. Intravenous maintenance fluids will be given at a rate of 4ml/kg/hour until the child is able to drink and retain oral fluids. Antipyretics, anticonvulsants and treatment for hypoglycaemia and other treatments will be given as clinically required and will be administered according to nationally agreed protocols. Children with Hb <4 g/dl (or Hb <6 g/dl and respiratory distress) will be transfused with 20mls/kg of whole blood as soon as blood is available. In the absence of blood standard care as per local treatment guidelines will be followed.

Table 2: Clinical monitoring

Table 2: Clinical monitoring

	Assessment Time									
**Procedure**	**Adm**	**1h**	**2h**	**4h**	**8h**	**9h**	**17h**	**24h**	**At least bi- daily until discharge**	**Day 7**	**Day 28**
Clinical assessment	X	X	X	X	X			X	X	X	X
ECG continuous to 24 hours	X							X			
APTT and coagulation tests (ACT) (1.7ml)	X	X 1 hr post dose				X		X 1 hr post dose			
Microperfusion[&] (Kilifi)	x					x					
Lactate (point of care)	X	X				X		X			
* Standard clinical test non-research (FBC, POC chemisty Istat)	X							X		X (Hb or FBC only)	
Malaria slides	X					X		X	36,48 (and 72 hr)	X	X
Stored red cells (Kilifi) & admission plasma **	X					X					X
Samples for PK studies		X				X					
Neuro-developmental exam/questionnaire	X										X

[&] Kilifi Site only

* Standard- non research clinical tests full blood count, clinical chemistry POC IStat (electrolytes, Ph BUN) will be done at admission and at 24 hours and Day 7 (Full blood count only).

Other laboratory test: venous blood gases (including base excess) will be done at 0, 9 and 24 hours

Malaria slide and morphology will be done at 0, 9, 17, 24, 36, 48 hours (and 72 hours if in hospital); at follow up (Day 7 and 28)

**For quantitative plasma HRP2 assessment, biomarkers and parasite morphology (Kilifi site only)

Clinical Assessment: Vital signs by bedside patient monitor (including temperature, heart rate, respiratory rate, blood pressure, oxygen saturation, conscious level). These will continue twice-daily after 24 hours until the child is discharged from HDU. All children will be managed on the HDU until conscious and able to take and retain oral fluids/food. During this time children will have 4 hours vital signs conducted. During the period of sevuparin administration (0-24 hours) children with have continuous electrocardiogram (ECG) monitoring for safety. However, it is notable that the trial in Thai adults showed no signs of QT prolongation [36]. Once discharged from HDU children will be reviewed daily until discharge and followed up at day 7 and day 28. On admission and Day 28 children will be assess by an adapted Kilifi Developmental Index to assess developmental status and clinically for neurodevelopmental sequelae **(see Table 2).**

Non-compliance is limited by the intervention being administered by clinical teams during admission. Any child who develops APTT>2.5xULN (grade 3 toxicity) will not receive further doses of sevuparin but will continue to be followed up. Children lost to follow-up before day 28 will be traced for vital status (permission requested within consent) using locator data and multiple contact phone numbers recorded before discharge.

Additional laboratory tests

At 1- and 8- hours and Day 28 plasma and red cell pellets will be used measure of the level Plasmodium falciparum Histidine Rich Protein (plasma); presence of rosetting and detailed microscopic examination of the infected red-cell morphology to stage the maturity of the parasite.

11 SAFETY REPORTING

The principles of ICH GCP require that both investigators and sponsors follow specific procedures when notifying and reporting adverse events or reactions in clinical trials.

DEFINITIONS

The definitions of the EU Directive 2001/20/EC Article 2 based on the principles of ICH GCP apply to this trial protocol. These definitions are given below.

TABLE	DEFINITION
Adverse Event (AE)	Any untoward medical occurrence in a patient or clinical trial subject to whom a medicinal product has been administered including occurrences that are not necessarilycaused by or related to that product.
Adverse Reaction (AR)	Any untoward and unintended response to an investigational medicinal product related to any doseadministered.
Unexpected Adverse Reaction (UAR)	An adverse reaction, the nature or severity of which is not consistent with the information about the medicinal product in question set out in the Summary of Product Characteristics (SPC) or Investigator Brochure (IB) for thatproduct.
Serious Adverse Event (SAE) or Serious Adverse Reaction (SAR) orSuspected Unexpected Serious Adverse Reaction (SUSAR)***	Respectively any adverse event, adverse reaction or unexpected adverse reaction that:<br>▪ Results in death<br>▪ Is life-threatening*<br>▪ Requires hospitalisation or prolongation of existing hospitalisation**<br>▪ Results in persistent or significant disability or incapacity<br>▪ Consists of a congenital anomaly or birth defect<br>▪ Is another important medical condition

*The term life-threatening in the definition of a serious event refers to an event in which the patient is at risk of death at the time of the event

**Hospitalisation is defined as an inpatient admission, regardless of length of stay, even if the hospitalisation is a precautionary measure for continued observation. Hospitalisations for a pre-existing condition (including

elective procedures that have not worsened) do not constitute an SAE.

Adverse events will be graded following the Common Toxicity Criteria v5.0 (https://ctep.cancer.gov/protocoldevelopment/electronic_applications/docs/CTCAE_v5_Quick_Reference_5x7.pdf). Any conditions not explicitly included should be graded according to the following definitions, which have been adapted from Directive 2001/20/EC of the European Parliament (Clinical Trials Directive) [61] and ICH-GCP guidelines (E6(R1), 1996) for grading of the severity of the events.

- Grade 1 Mild; asymptomatic or mild symptoms; clinical or diagnostic observations only; intervention not indicated.
- Grade 2 Moderate; minimal, local or noninvasive intervention indicated; limiting age-appropriate instrumental activities of daily living.
- Grade 3 Severe or medically significant but not immediately life-threatening; hospitalization or prolongation of hospitalization indicated; disabling; limiting self care activities of daily living.
- Grade 4 Life-threatening consequences; urgent intervention indicated.
- Grade 5 Death related to AE.

11.1.1 Exempted Adverse Events

All patients eligible for SEVUSMAART are critically ill and due to the complexity of their condition are at increased risk of experiencing AEs. Many of these events are expected as a result of the patient's underlying severe malaria and are not related to participation in the trial. Consequently, AEs occurring as a result of the patient's medical condition will not be reported eg episode of hypoglycaemia or seizures. Pre-existing conditions do not qualify as AEs unless they worsen in a way that is not expected for the patient's medical condition at admission but should be documented in the patient's medical notes.

Other than this, Adverse Events include:

- An exacerbation of a pre-existing illness
- An increase in frequency or intensity of a pre-existing episodic event or condition
- A condition (even though it may have been present prior to the start of the trial) detected after trial drug administration
- Continuous persistent disease or a symptom present at baseline that worsens following administration of the study treatment

Adverse Events do not include:

- Medical or surgical procedures; the condition that leads to the procedure is the adverse event
- Pre-existing disease or a condition present before treatment that does not worsen
- Hospitalisations where no untoward or unintended response has occurred, eg, elective cosmetic surgery, social admissions
- Overdose of medication without signs or symptoms

CAUSALITY

The assignment of causality should be made by the investigator responsible for the care of the participant using the definitions in the table below. If any doubt about the causality exists the local investigator should inform the study coordination centre who will notify the Principal Investigator. Other clinicians may be asked to advise in some cases.

In the case of discrepant views on causality between the investigator and others, all parties will discuss the case.

Relationship	Description
**Unrelated**	There is no evidence of any causal relationship
**Unlikely**	There is little evidence to suggest there is a causal relationship (e.g. the event did not occur within a reasonable time after administration of the trial medication). There is another reasonable explanation for the event (e.g., the participant's clinical condition, other concomitant treatment).
**Possible**	There is some evidence to suggest a causal relationship (e.g., because the eventoccurs within a reasonable time after administration of the trial medication).<br>However, the influence of other factors may have contributed to the event (e.g.,the participant's clinical condition, other concomitant treatments).
**Probable**	There is evidence to suggest a causal relationship and the influence of other factors is unlikely.
**Definitely**	There is clear evidence to suggest a causal relationship and other possible contributing factors can be ruled out.
**Not assessable**	There is insufficient or incomplete evidence to make a clinical judgement of the causal relationship.

11.2 Reporting Procedures

At each clinical review the clinician or nurse will check for potential SAEs and grade 3 or 4 AEs, and any potentially intervention-related AEs (e.g., coagulation test results). All serious adverse events will be reported in the case report form (CRF) and on SAE forms. All grade 3 or 4 events (regardless of causality) will be reported on the CRFs. Coagulation adverse events will be identified directly from the laboratory values reported on the laboratory test result CRF. The reporting procedure is captured within the safety reporting Standard Operating Procedure (SOP). Any questions concerning adverse event reporting should be directed to the study coordination centre in the first instance.

SAEs will be reported to the study coordinator, using an SAE form. The SAE form asks for nature of event, date of onset, severity, corrective therapies given, outcome and causality (i.e., unrelated, unlikely, possible, probably, definitely) to all medical interventions received. The responsible investigator should sign the causality of the event. Additional information should be sent within 5 daysif the reaction has not resolved at the time of reporting. The SAEs will be reported by the study PI to ethics and regulatory bodies within 24 hours(electronically) and 5 days for the full report.

12 DATA MANAGEMENT

12.1 Data Storage

All clinical and laboratory data will be recorded in the CRF and stored with a unique serial number identifier. Data will be entered onto Open Clinica. All data will be regularly backed up and backup copies stored both on and off site. Paper records will be archived in locked cabinets at Kilifi County Hospital, Kenya. These cabinets will have limited access with prior authorisation. All data will be partially- anonymized prior to presentation or publication of any results. Archive documents will be sent for long term storage (10 years) at an appropriate facility according to Kenya Medical Research Institute (KEMRI)policies.

12.2 Data Management and Statistical Analysis

Clinical data will be summarized using means and medians where appropriate for continuous data depending on the distribution. Primary and secondary endpoints will be described using means or medians or proportions. Analyses will follow intention-to-treat. As this is a Phase I trial no subgroup analyses are planned.

12.3 Intellectual Property

Any Intellectual property rights that arise from the work will be safeguarded according to the KEMRI IPR Policy of 2015 and the Industrial Property Act of 2001, sections 32, 58 and 80. The scientific and intellectual contributions of all persons involved in the research will be appropriately acknowledged in all publications and presentations arising from the work.

12.4 Time Frame/Duration of the Project:

a) Study enrolment – 15 months total, 1st September 2022 – 31st December 2023
b) Data analysis – 1st February 2024 – 1st May 2024 – 3 months
c) Report preparation – 1st May 2024 – 1st July 2024 – 2 months

13 ETHICAL CONSIDERATION:

Ethical approval will be sought from KEMRI Scientific and Ethics Review Unit (SERU) and from Imperial College Research Ethics Committee (ICREC), who is the sponsor of the study.

13.1 Human subjects

13.1.1 Risks

"First, do no harm." The study will be performed in patients who may potentially benefit from the treatment. The risks of cannula insertion and blood drawing include pain, infection at the site of the cannula and thrombophlebitis. These will be minimised by careful technique according to a standard SOP, cannula site inspection and replacement or removal where necessary. No more than 1ml/kg of blood will be drawn for research at any one time. The trial will be recruiting patients with severe illness and likely a high mortality rate. At the start of the trial, the site will receive appropriate training on the use of Sevuparin and will have 2 dedicated clinicians. Sevuaprin can lead to minimal and non-clinically relevant changes in APPT (grade II toxicity) In the Phase I dose escalation trial, sevuparin was found to be safe and well-tolerated with minimal effects on APTT at doses of 1.5 mg/kg and 3mg/kg every six hours but a dose of 6.0 mg/kg led to higher APTT values. The Data Monitoring Committee (DMC) therefore recommended that the study should continue to the Phase II with a dose of 3.0 mg/kg sevuparin every 6h, given the potentially increased risk of adverse events at higher doses.

Risk will be minimised by the trial design/methods (see page 23) whereby the initial participants (two 'cohorts' of 2 children each, i.e. 4 children in total) will receive a dose of 1.5 mg/kg/dose with the plan to escalate up to a cohort of 2 children receiving 3mg/kg/dose and a cohort of two children receiving 6.0 mg/kg/dose (maximum). In order to determine whether, and the rate of escalation, to a higher dosage for subsequent patients after the first 4 children we will use a design called the Continuous Reassessment Method (CRM). This starts with an estimated dose-toxicity curve, reflecting the probability of experiencing a toxicity event (here APTT>2.5xULN 1h after any of the three doses) to the dose received. In this method of dose finding, the dose-toxicity curve is fitted to the data after each child is enrolled, based on their observed dose and toxicity outcome. After the first 8 children, each subsequent patient would be assigned the largest dose with an estimated risk of toxicity below the target toxicity level, designated as the maximum tolerated dose (MTD).

13.1.2 Benefits

All patients will be closely monitored so that clinical deteriorations can be identified at the earliest opportunity and appropriate therapy initiated. In general, the high dependency ward at Kilifi County Hospital has considerable experience with this population and this will serve to minimise the risks to the patients and the trial. Prior to the start, the dedicated study teams will undergo detailed training on general management of severe malaria and its complications and receive very detailed training on the use of sevuparin. We believe this will afford all children enrolled in the trial with a higher quality of care.

All routine non-trial medications required by the hospital to treat the child will be made available.

Hospital bills for participants older than 5 years will be covered by the study (covering the costs for standard treatment for severe malaria and related complications).

The parents or guardians for the children will be asked to return for a follow up clinic visit at day 7 and day 28 and thus will be offered continuing care for concurrent illness, including any investigations or blood tests that are clinically indicated.

13.2 Patient Consent

Prospective written, informed consent will be sought from parents or guardians of children who are considered to be sufficiently stable. Parents or guardians will be given an information sheet in their usual language containing details of the SEVUSMAART study. These will be translated into to local languages, and then back translated (to ensure details are correct) prior to piloting before the initiation of the trial. The sheet will be read aloud to those who are unable to read. Parents and guardians will be encouraged to ask questions about the trial prior to signing the consent form. Consent will include permission for the collection of data and for aetiological investigations. The rights of the participant to refuse to participate without giving reasons must be respected. A number of children will present as emergencies where delay in study enrolment, and thus treatment, will not be practical or indeed humane. We will use a modified form of deferred consent that we developed, received ethical approval for, and used in the FEAST trial. It proposes to use a 'two-stage' consent process in this circumstance [57]. Verbal assent will be sought from parents or guardians by the admitting medical team, if it is considered that the full consent process would significantly delay enrolment, and consequently could be detrimental to the child's health. Full consent will be sought once the child's clinical condition has been stabilized. Caregivers will be provided with a brief verbal description of the trial and will be given the opportunity to "opt out" of clinical research. The clinician will then sign the verbal assent form, which will be filed with the consent form. A social science study of the consent processes used in FEAST found this to be acceptable to parents and health-care workers [62]. As in the FEAST trial, if following an assent process a child died prior to full written consent, full consent would not be sought. This was process of emergency consent was approved (by multiple ethics research committees) for FEAST and has been subsequently approved for use in a transfusion trial in Uganda and Blantyre (TRACT) [58].

13.3 WITHDRAWAL OF PATIENTS and Protocol Treatment Discontinuation

In consenting to the trial, patients are consenting to trial treatment (sevuparin), data collection and follow-up. If a carer wishes to withdraw their child from trial treatment, the investigator will explain the importance and benefits of follow-up, and the value of allowing routine clinical data to be used for trial purposes. Patients/parents are free to discontinue any part of their trial treatment or discontinue from follow up. After the participant has entered the trial, the clinician remains free to give alternative treatment to that specified in the protocol at any stage if he/she feels it is in the participant's best interest, but the reasons for doing so should be recorded. In these cases, the participants remain within the study for the purposes of follow-up and data analysis. All carers and participants are free to withdraw at any time the protocol treatment without giving reasons and

without prejudicing further treatment. If they do not wish to remain on trial follow-up, however, their decision must be respected, and the patient will be withdrawn from the trial completely.

13.4 Protocol Treatment Discontinuation

An individual patient may stop treatment early or be stopped early for any of the following reasons:

- Unacceptable toxicity or adverse event
- Intercurrent illness that prevents further treatment
- Any change in the patient's condition that justifies the discontinuation of treatment in the clinician's opinion
- Withdrawal of consent for treatment by the patient or parent.

Participation in the trial is entirely voluntary, and parents, carers or older children may choose to discontinue the trial treatment at any time without penalty or loss of benefits to which they are otherwise entitled. Although not required to give a reason for discontinuing their trial treatment, a reasonable effort should be made to establish this reason while fully respecting the patient's rights.

Patients should remain in the trial for the purpose of follow-up wherever possible (unless the patient withdraws their consent for follow-up). If a patient withdraws from the trial, the medical data collected during their previous consented participation in the trial will be kept and used in analysis. This will also apply to parents/carers who withdraw from the trial after assent, that have not completed the deferred consent process. Consent for future use of stored samples already collected can be refused when leaving the trial early (but this should be discouraged and should follow a discussion). If consent for future use of stored samples already collected is refused, then all such samples will be destroyed following Kenya Medical Research Institute (KEMRI) policies.

13.5 Community Engagement Strategy and feedback

Community engagement will be through regular meetings with the community involving KEMRI-Community Representatives and County Health teams. At these meetings, information and feedback will be given and received. Information arising from the study will be fed back through hospital-wide meetings. This is a Phase I trial of an emergency intervention where our engagement has been at a scientific rather than public/community level. If a larger platform trial arose from this study, we aim to develop a dedicated and informed engagement strategy as part of this future trial. In general, we plan to feed into existing community engagement mechanisms. We aim to build general community awareness of research processes at the local hospital, and support community representative inputs into decisions around research design, consent procedures, patient information and trial conduct.

13.6 Compensation

Families will not incur any costs from participation in this study. All children who are enrolled in the study travel expenses for attending the visits on Day 7 and Day 28 will be paid, based on the cost of public transport to and from the participant's home and compensation for out-of-pocket expenses using standard rates (KES 350). At follow up visits snacks and drinks will be provided for children enrolled in the trial.

13.7 Confidentiality

Participant's identification data will be required for the registration process. All clinical and laboratory data will be recorded in the CRF and stored with a unique serial number identifier. Information will only be made available to those caring for the child and those directly involved with the study. Data will be entered onto Open Clinica (FDA approved, web-based application). All data will be regularly backed up and backup copies stored both on and off site. Paper records will be archived in locked cabinets. These cabinets will have limited access with prior authorisation. All data will be partially-anonymized prior to presentation or publication of any results. All clinical data will be held confidentially, and personal identifiers will be removed before analysis of the data and presentation of the results.

13.8 Data Sharing

After completion of the study, requests for data access from researchers outside the study team will be considered by a subgroup of the Centre Scientific Committee (Data Governance Committee), and where indicated, requestors will be asked to develop scientific protocols for approval of secondary analyses. The potential to share data will be included in the participant Information and Consent Form.

13.9 Indemnity

Imperial College London holds negligent harm and non-negligent harm insurance policies, which apply to this study.

13.10 Sponsor

Imperial College London will act as the main Sponsor for this study and delegates this responsibility to the KWTRP, Kilifi and MRC CTU at UCL to oversee the implementation of the study by ensuring that arrangements are put into place for adequate management, monitoring, analysis and reporting of the trial.

EXPECTED APPLICATION OF THE RESULTS

The results of the Phase I trial will identify the final dosage selected for the Phase II that will include both efficacy and safety outcomes. The primary endpoint for the future Phase II reflects the primary hypothesis that sevuparin improves microcirculatory flow by reversing and preventing parasite sequestration. Data collected in the Phase I trial will assess whether the planned primary endpoint for the Phase II trial, lactate clearance at 8 hours, is realistic and feasible.

TIMEFRAME

Total duration of 15 months:
Start Date 1st September 2022
Recruitment over 15 Months (3 malaria seasons)
Data Analysis and report writing 2 months

14 R EFERENCES

1. Snow RW, Sartorius B, Kyalo D, Maina J, Amratia P, Mundia CW, Bejon P, Noor AM: **The prevalence of Plasmodium falciparum in sub-Saharan Africa since 1900**. *Nature* 2017, **550**(7677):515-518.
2. Noor AM, Kinyoki DK, Mundia CW, Kabaria CW, Mutua JW, Alegana VA, Fall IS, Snow RW: **The changing risk of Plasmodium falciparum malaria infection in Africa: 2000–10: a spatial and temporal analysis of transmission intensity**. *The Lancet* 2014, **383**(9930):1739-1747.
3. Alonso P, Noor AM: **The global fight against malaria is at crossroads**. *Lancet* 2017, **390**(10112):2532-2534.
4. Organization WH: **World Malaria Report**. In. Geneva; 2017.
5. Dondorp AM, Fanello CI, Hendriksen IC, Gomes E, Seni A, Chhaganlal KD, Bojang K, Olaosebikan R, Anunobi N, Maitland K *et al*: **Artesunate versus quinine in the treatment of severe falciparum malaria in African children (AQUAMAT): an open-label, randomised trial**. *Lancet* 2010, **376**(9753):1647-1657.
6. Maitland K: **Management of severe paediatric malaria in resource-limited settings**. *BMC Med* 2015, **13**(1):42.
7. Maitland K: **Severe Malaria in African Children - The Need for Continuing Investment**. *N Engl J Med* 2016, **375**(25):2416-2417.
8. Okebe J, Eisenhut M: **Pre-referral rectal artesunate for severe malaria**. *Cochrane Database Syst Rev* 2014(5):CD009964.
9. John CC, Kutamba E, Mugarura K, Opoka RO: **Adjunctive therapy for cerebral malaria and other severe forms of Plasmodium falciparum malaria**. *Expert Rev Anti Infect Ther* 2010, **8**(9):997-1008.
10. von Seidlein L, Olaosebikan R, Hendriksen IC, Lee SJ, Adedoyin OT, Agbenyega T, Nguah SB, Bojang K, Deen JL, Evans J *et al*: **Predicting the clinical outcome of severe falciparum malaria in african children: findings from a large randomized trial**. *Clinical infectious diseases : an official publication of the Infectious Diseases Society of America* 2012, **54**(8):1080-1090.
11. White NJ, Turner GD, Day NP, Dondorp AM: **Lethal malaria: Marchiafava and Bignami were right**. *The Journal of infectious diseases* 2013, **208**(2):192-198.
12. Kyes S, Horrocks P, Newbold C: **Antigenic variation at the infected red cell surface in malaria**. *Annu Rev Microbiol* 2001, **55**:673-707.
13. Chen Q, Schlichtherle M, Wahlgren M: **Molecular aspects of severe malaria**. *Clin Microbiol Rev* 2000, **13**(3):439-450.
14. Dondorp AM, Kager PA, Vreeken J, White NJ: **Abnormal blood flow and red blood cell deformability in severe malaria**. *Parasitol Today* 2000, **16**(6):228-232.
15. Dondorp AM, Ince C, Charunwatthana P, Hanson J, van Kuijen A, Faiz MA, Rahman MR, Hasan M, Bin Yunus E, Ghose A *et al*: **Direct in vivo assessment of microcirculatory dysfunction in severe falciparum malaria**. *The Journal of infectious diseases* 2008, **197**(1):79-84.
16. Brown HT, G Rogerson, SJ Tembo, M Mwenechanya, J Dzinjalamala, F Molyneux, ME Taylor, TE.: **Cytokine expression in the brain in human cerebral malaria.** *J Infect Dis* 1999, **180**:1742-1746.
17. Hanson J, Lam SW, Mahanta KC, Pattnaik R, Alam S, Mohanty S, Hasan MU, Hossain A, Charunwatthana P, Chotivanich K *et al*: **Relative contributions of macrovascular and microvascular dysfunction to disease severity in falciparum malaria**. *The Journal of infectious diseases* 2012, **206**(4):571-579.

18. Ishioka H, Ghose A, Charunwatthana P, Maude R, Plewes K, Kingston H, Intharabut B, Woodrow C, Chotivanich K, Sayeed AA *et al*: **Sequestration and Red Cell Deformability as Determinants of Hyperlactatemia in Falciparum Malaria**. *The Journal of infectious diseases* 2016, **213**(5):788-793.
19. Jeeyapant A, Kingston HW, Plewes K, Maude RJ, Hanson J, Herdman MT, Leopold SJ, Ngernseng T, Charunwatthana P, Phu NH *et al*: **Defining Surrogate Endpoints for Clinical Trials in Severe Falciparum Malaria**. *PloS one* 2017, **12**(1):e0169307.
20. Aramburo A, Todd J, George EC, Kiguli S, Olupot-Olupot P, Opoka RO, Engoru C, Akech SO, Nyeko R, Mtove G *et al*: **Lactate clearance as a prognostic marker of mortality in severely ill febrile children in East Africa**. *BMC medicine* 2018, **16**(1):37.
21. Ponsford MJ, Medana IM, Prapansilp P, Hien TT, Lee SJ, Dondorp AM, Esiri MM, Day NP, White NJ, Turner GD: **Sequestration and microvascular congestion are associated with coma in human cerebral malaria**. *The Journal of infectious diseases* 2012, **205**(4):663-671.
22. Hendriksen IC, White LJ, Veenemans J, Mtove G, Woodrow C, Amos B, Saiwaew S, Gesase S, Nadjm B, Silamut K *et al*: **Defining falciparum-malaria-attributable severe febrile illness in moderate-to-high transmission settings on the basis of plasma PfHRP2 concentration**. *The Journal of infectious diseases* 2013, **207**(2):351-361.
23. Angeletti D, Sandalova T, Wahlgren M, Achour A: **Binding of subdomains 1/2 of PfEMP1-DBL1alpha to heparan sulfate or heparin mediates Plasmodium falciparum rosetting**. *PloS one* 2015, **10**(3):e0118898.
24. Barragan A, Fernandez V, Chen Q, von Euler A, Wahlgren M, Spillmann D: **The duffy-binding-like domain 1 of Plasmodium falciparum erythrocyte membrane protein 1 (PfEMP1) is a heparan sulfate ligand that requires 12 mers for binding**. *Blood* 2000, **95**(11):3594-3599.
25. Munir M, Husada T, Rampengan TH, Mustadjab I, Wulur FH: **Heparin in the treatment of cerebral malaria (a preliminary report)**. *Paediatrica Indonesiana* 1976, **16**(11-12):489-495.
26. Munir M, Tjandra H, Rampengan TH, Mustadjab I, Wulur FH: **Heparin in the treatment of cerebral malaria**. *Paediatrica Indonesiana* 1980, **20**(1-2):47-50.
27. Hemmer CJ, Kern P, Holst FG, Nawroth PP, Dietrich M: **Neither heparin nor acetylsalicylic acid influence the clinical course in human Plasmodium falciparum malaria: a prospective randomized study**. *The American journal of tropical medicine and hygiene* 1991, **45**(5):608-612.
28. Reid HA, Sucharit P: **Ancrod, heparin, and -aminocaproic acid in simian Knowlesi malaria**. *Lancet* 1972, **2**(7787):1110-1112.
29. Reid HA: **Letter: Adjuvant treatment of severe falciparum malaria, intravascular coagulation, and heparin**. *Lancet* 1975, **1**(7899):167-168.
30. Carlson J, Ekre HP, Helmby H, Gysin J, Greenwood BM, Wahlgren M: **Disruption of Plasmodium falciparum erythrocyte rosettes by standard heparin and heparin devoid of anticoagulant activity**. *The American journal of tropical medicine and hygiene* 1992, **46**(5):595-602.
31. Kulane A, Ekre HP, Perlmann P, Rombo L, Wahlgren M, Wahlin B: **Effect of different fractions of heparin on Plasmodium falciparum merozoite invasion of red blood cells in vitro**. *The American journal of tropical medicine and hygiene* 1992, **46**(5):589-594.
32. Leitgeb AM, Blomqvist K, Cho-Ngwa F, Samje M, Nde P, Titanji V, Wahlgren M: **Low anticoagulant heparin disrupts Plasmodium falciparum rosettes in fresh clinical isolates**. *The American journal of tropical medicine and hygiene* 2011, **84**(3):390-396.
33. **Patent PCT 64734PC2**. In.
34. Saiwaew S, Sritabal J, Piaraksa N, Keayarsa S, Ruengweerayut R, Utaisin C, Sila P, Niramis R, Udomsangpetch R, Charunwatthana P *et al*: **Effects of sevuparin on rosette formation and cytoadherence of Plasmodium falciparum infected erythrocytes**. *PloS one* 2017, **12**(3):e0172718.
35. **Severe malaria**. *Trop Med Int Health* 2014, **19 Suppl 1**:7-131.

36. Leitgeb AM, Charunwatthana P, Rueangveerayut R, Uthaisin C, Silamut K, Chotivanich K, Sila P, Moll K, Lee SJ, Lindgren M *et al*: **Inhibition of merozoite invasion and transient de-sequestration by sevuparin in humans with Plasmodium falciparum malaria**. *PloS one* 2017, **12**(12):e0188754.
37. Esmon C: **Blood Coagulation**. In: *Hematology of Infancy and Childhood.* edn. Edited by Orkin S. PhiladelphiA: W.B. Saunders; 1998: 1531-1552.
38. Esmon CT, Fukudome K, Mather T, Bode W, Regan LM, Stearns-Kurosawa DJ, Kurosawa S: **Inflammation, sepsis, and coagulation**. *Haematologica* 1999, **84**(3):254-259.
39. Turner GD, Ly VC, Nguyen TH, Tran TH, Nguyen HP, Bethell D, Wyllie S, Louwrier K, Fox SB, Gatter KC *et al*: **Systemic endothelial activation occurs in both mild and severe malaria. Correlating dermal microvascular endothelial cell phenotype and soluble cell adhesion molecules with disease severity**. *Am J Pathol* 1998, **152**(6):1477-1487.
40. Boehme MW, Werle E, Kommerell B, Raeth U: **Serum levels of adhesion molecules and thrombomodulin as indicators of vascular injury in severe Plasmodium falciparum malaria**. *The Clinical investigator* 1994, **72**(8):598-603.
41. Hemmer CJ, Bierhaus A, von Riedesel J, Gabat S, Liliensiek B, Pitronik P, Lin J, Grauer A, Amiral J, Ziegler R *et al*: **Elevated thrombomodulin plasma levels as a result of endothelial involvement in plasmodium falciparum malaria**. *Thromb Haemost* 1994, **72**(3):457-464.
42. Gerardin P, Rogier C, Ka AS, Jouvencel P, Brousse V, Imbert P: **Prognostic value of thrombocytopenia in African children with falciparum malaria**. *The American journal of tropical medicine and hygiene* 2002, **66**(6):686-691.
43. Ladhani S, Lowe B, Cole AO, Kowuondo K, Newton CR: **Changes in white blood cells and platelets in children with falciparum malaria: relationship to disease outcome**. *British journal of haematology* 2002, **119**(3):839-847.
44. Schmidt D, Gyr K: **Malaria at the University Hospital and the St.-Clara Hospital, Basel, in the period of 1970-1979**. *Bulletin de la Societe de pathologie exotique et de ses filiales* 1983, **76**(5):486-492.
45. Pukrittayakamee S, White NJ, Clemens R, Chittamas S, Karges HE, Desakorn V, Looareesuwan S, Bunnag D: **Activation of the coagulation cascade in falciparum malaria**. *Transactions of the Royal Society of Tropical Medicine and Hygiene* 1989, **83**(6):762-766.
46. Clemens R, Pramoolsinsap C, Lorenz R, Pukrittayakamee S, Bock HL, White NJ: **Activation of the coagulation cascade in severe falciparum malaria through the intrinsic pathway**. *British journal of haematology* 1994, **87**(1):100-105.
47. Hemmer CJ, Kern P, Holst FG, Radtke KP, Egbring R, Bierhaus A, Nawroth PP, Dietrich M: **Activation of the host response in human Plasmodium falciparum malaria: relation of parasitemia to tumor necrosis factor/cachectin, thrombin-antithrombin III, and protein C levels**. *The American journal of medicine* 1991, **91**(1):37-44.
48. Marsh K, Forster D, Waruiru C, Mwangi I, Winstanley M, Marsh V, Newton C, Winstanley P, Warn P, Peshu N *et al*: **Indicators of life-threatening malaria in African children**. *The New England journal of medicine* 1995, **332**(21):1399-1404.
49. Reid HA, Nkrumah FK: **Fibrin-degradation products in cerebral malaria**. *Lancet* 1972, **1**(7744):218-221.
50. Francischetti IM, Seydel KB, Monteiro RQ, Whitten RO, Erexson CR, Noronha AL, Ostera GR, Kamiza SB, Molyneux ME, Ward JM *et al*: **Plasmodium falciparum-infected erythrocytes induce tissue factor expression in endothelial cells and support the assembly of multimolecular coagulation complexes**. *J Thromb Haemost* 2007, **5**(1):155-165.
51. Akingbola TS, Shokunbi WA, Olumese PE: **Coagulation profile in Nigerian children with cerebral malaria**. *The Nigerian postgraduate medical journal* 2006, **13**(3):195-199.
52. Moxon CA, Wassmer SC, Milner DA, Jr., Chisala NV, Taylor TE, Seydel KB, Molyneux ME, Faragher B, Esmon CT, Downey C *et al*: **Loss of endothelial protein C receptors links**

coagulation and inflammation to parasite sequestration in cerebral malaria in African children. *Blood* 2013, **122**(5):842-851.
53. Andrew M, Vegh P, Johnston M, Bowker J, Ofosu F, Mitchell L: **Maturation of the hemostatic system during childhood**. *Blood* 1992, **80**(8):1998-2005.
54. Garrett-Mayer E: **The continual reassessment method for dose-finding studies: a tutorial**. *Clin Trials* 2006, **3**(1):57-71.
55. Onar A, Kocak M, Boyett JM: **Continual reassessment method vs. traditional empirically based design: modifications motivated by Phase I trials in pediatric oncology by the Pediatric Brain Tumor Consortium**. *J Biopharm Stat* 2009, **19**(3):437-455.
56. Niederdockl J, Dempfle CE, Schonherr HR, Bartsch A, Miles G, Laggner A, Pathil A: **Point-of-care PT and aPTT in patients with suspected deficiencies of coagulation factors**. *Int J Lab Hematol* 2016, **38**(4):426-434.
57. Maitland K, Molyneux S, Boga M, Kiguli S, Lang T: **Use of deferred consent for severely ill children in a multi-centre phase III trial**. *Trials* 2011, **12**:90.
58. Mpoya A, Kiguli S, Olupot-Olupot P, Opoka RO, Engoru C, Mallewa M, Chimalizeni Y, Kennedy N, Kyeyune D, Wabwire B *et al*: **Transfusion and Treatment of severe anaemia in African children (TRACT): a study protocol for a randomised controlled trial**. *Trials* 2015, **16**(1):593.
59. Maitland K, Kiguli S, Opoka R, Olupot-Olupot P, Engoru C, Njuguna P, Bandika V, Mpoya A, Bush A, Williams T *et al*: **Children's Oxygen Administration Strategies Trial (COAST): A randomised controlled trial of high flow versus oxygen versus control in African children with severe pneumonia**. *Wellcome Open Res* 2017, **2**:1-29.
60. Abubakar A, Holding P, van Baar A, Newton CR, van de Vijver FJ: **Monitoring psychomotor development in a resource-limited setting: an evaluation of the Kilifi Developmental Inventory**. *Ann Trop Paediatr* 2008, **28**(3):217-226.
61. Union EPaCoE: **Directive 2001/20/EC of European Parliament and of the Council of 4 April 2001 on the approximation of the laws, regulations and administrative provisions of the Member States relating to the implementation of good clinical practice in the conduct of clinical trials on medicinal products for human use**. *Official Journal L121* 2001.
62. Molyneux S, Njue M, Boga M, Akello L, Olupot-Olupot P, Engoru C, Kiguli S, Maitland K: **'The words will pass with the blowing wind': staff and parent views of the deferred consent process, with prior assent, used in an emergency fluids trial in two African hospitals**. *PLoS One* 2013, **8**(2):e54894.

15 ROLE OF INVESTIGATORS

Name	Study design and development	Professional and Community engagement	Clinical care	Data and sample collection	Endpoint review	Clinical data Analysis	Report writing
Kathryn Maitland	⊙		⊙	⊙			⊙
Mainga Hamaluba	⊙	⊙	⊙	⊙			⊙
Mike Chaponda			⊙	⊙			⊙
Nchafatso Obonyo			⊙	⊙			⊙
Emmanual Odugu		⊙		⊙	⊙		⊙
Tom Williams	⊙		⊙	⊙			⊙
Walker, George and Gibb	⊙				⊙	⊙	⊙
Andrew Turnbull	⊙		⊙	⊙			⊙
Day, Dorndorp	⊙					⊙	⊙

Appendices

1) Patient information sheets, consent forms, assent form, withdrawal form

Docusign Envelope ID: AB5F25C1-23D2-4CE0-B871-54E4507AD149

Funders wellcome

Sponsors Imperial College London

SEVUparin as a potential Adjunctive Treatment in children with severe malaria

(SEVUSMART trial)

Statistical Analysis Plan			
Version Number and Date: Draft 1.0 11th December 2025<br>Supersedes version: Draft 0.4 10th December 2025			
**Author**	**Position**	**Signature**	**Date**
Elizabeth George	Trial Statistician	Signed by: Elizabeth George 13936A7CF0D2430...	11-Dec-2025
**Approved by**			
Professor A. Sarah Walker	Co-Trial Statistician	Signed by: Ann Sarah Walker A21A8226533E49A...	11-Dec-2025
Professor Kathryn Maitland	Chief Investigator	DocuSigned by: Kath Maitland 4FEBBA0BB1BD4CC...	11-Dec-2025

Revision History

Version	Author	Date	Reason for Revision
Draft 0.2	Elizabeth George	18/06/2020	First draft based on protocol version 1.0
Draft 0.3	Elizabeth George	5/11/2025	Updated for review
Draft 0.4	Elizabeth George	10/12/2025	Updated following review by AS Walker and K Maitland
V1.0	Elizabeth George	11/12/2025	Up versioned for signatures

1) Trial Design

Trial Outline

The objective of this trial is to identify the maximum tolerated dose (MTD) of intravenous sevuparin as an adjunctive therapy in children with severe malaria given as three infusions at 0, 8 and 16 hours using the Continual Reassessment Method (CRM) to adapt or inform subsequent doses for each child entering the trial, based on a toxicity event defined as any APTT >2.5 upper limit of normal (ULN) 1 hour after each dose, and updating the dose-toxicity model using the previous patients' APTT results.

Trial Population

Inclusion criteria

1. Aged between 3 months and 12 years admitted to the paediatric wards within the last 24h
2. Current or recent evidence of *P. falciparum* malaria (slide or rapid diagnostic test (RDT) positive)
3. Clinical evidence of severe malaria: impaired consciousness (coma (inability to localize painful stimulus) or prostration (inability to sit unsupported for those above 6 months)) or deep breathing
4. Lactate > 2 mmol/L
5. Guardian or parent willing and able to provide consent

Exclusion criteria

1. Clinical evidence or a history of a bleeding/coagulation disorder
2. A comorbidity which clinician believes has a significant risk of poor outcome e.g. malignancy, end-stage renal failure, major cardiac condition
3. Thrombocytopenia (platelet count <25 x10^9/L).

2) Interim reviews

The DMC will review data after the first 2 'cohorts' of 2 children each have been enrolled to the lowest dose, and then after each Dose Limiting Toxicity event. The continual reassessment method uses data from each enrolled participant to update the dose-toxicity curve and then suggests an escalation of dose to the largest dose with risk of estimated risk of toxicity below the target toxicity level if appropriate.

Docusign Envelope ID: AB5F25C1-23D2-4CE0-B871-54E4507AD149

3) Endpoints

Primary Endpoint

- APTT>2.5xULN 1h post any sevuparin dose (grade 3 following the CTC)

Secondary Endpoints

Efficacy

- Change in lactate from 0 to 8 hours
- Presence of mature infected erythrocytes on the blood films at 8 and 24 hours
- Parasite clearance time
- If available: change in sublingual microcirculation over time

Safety Endpoints

- APTT 24h post enrolment (absolute level and grade)
- Development of abnormalites of coagulation indices (prothrombin) (Grade 2 and above)
- Neurological sequelae through day 28
- Mortality through day 28
- Serious adverse events through day 28
- Grade 3/4 adverse events through day 28

APTT 24h post enrolment will be used as an assessment of normalization 8h after the final sevuparin dose. Both absolute levels and grade will be considered.

De-novo evidence of neurological sequelae will be ascertained using a modified Kilifi Developmental Index [60] assessed at admission (to identify pre-exisiting conditions) and follow up (which we have adapted to use for the COAST trial) [59].

Serious adverse events will use the standardized definitions (see section 11 below). SAEs will be independently reviewed in real-time by the DMC.

Adverse events will be graded following the Common Toxicity Criteria v5.0 (https://ctep.cancer.gov/protocoldevelopment/electronic_applications/docs/CTCAE_v5_Quick_Reference_5x7.pdf).

Prothrombin time was included in the protocol but only measured on the first patient and not measured on further participants. This was due to a review of processes and prothrombin was deemed not relevant so analysis of this is not included in this SAP.

Docusign Envelope ID: AB5F25C1-23D2-4CE0-B871-54E4507AD149

4) Derivation of data to be analysed

Time
Time will be from enrolment for primary analysis and secondary analysis.

Definition of baseline
Baseline values for all measurements will be those recorded at screening either on the screening form, the baseline clinical evaluation form or their first blood test taken within 48 hours of admission as appropriate.

Definition of visit schedule
Analyses of measurements at a given point in follow up (clinical symptoms and neurological sequelae assessments) will use the closest available measurement to that time point in evenly spaced windows. If there are two measurements that are equally close to the timepoint, the earliest measurement will be used.

Definition of censoring
Children lost to clinic follow up will be censored on the date they were last known alive including data from contact tracing visits. For analyses concerning events at specific time points, surviving children will be censored at that time point. That is for analyses at 28 days, censoring will occur on day 28.

5) Statistical Analysis

Clinical data will be summarised using means and medians where appropriate for continuous data depending on the distribution. Primary and secondary endpoints will be described using means or medians or proportions.

All children with APTT measured will be included in the final analyses as only those children contributed to the continual reassessment model to identify the maximum tolerated dose.

An additional analysis will describe any child enrolled without APTT measured.

As this is a Phase I trial no subgroup analyses are planned.

All analysis will be included in the final report, but only analysis in bold below will be included in the DMC report.

Baseline characteristics

- **Sex: n(%) male, female**
- **Age at admission (months): median (IQR)**

Docusign Envelope ID: AB5F25C1-23D2-4CE0-B871-54E4507AD149

- **Weight (kg): median (IQR)**
- **Heart rate (bpm), axillary temperature (˚C), systolic blood pressure (mmHg), diastolic blood pressure (mmHg), oxygen saturation (%), respiratory rate (brpm), capillary refill time (s): median (IQR)**
- **Blantyre Coma Score (BCS): n (%) each value of score (0 to 5)**
- **Temperature gradient, weak pulse: n(%) yes, no**

Clinical history of this illness

- History of fever within 14 days, history of fever more than 14 days, history of cough, increased work of breathing, vomiting, inability to sit up right unsupported, diarrhoea, fits in this illness: n(%) yes, no, don't know
- Bloody diarrhoea: n(% of those with diarrhoea), yes, no, don't know
- **Seizures in this illness: n (%) yes, no, don't know**
- **Seizures lasting more than 30 minutes: n(%) yes, no, don't know**
- Haemoglobinuria: n(%) yes, no, don't know; median (IQR) length (days)
- Inability to sit upright unsupported (prostrate): n(%), yes, no

Treatment in this illness

- Admitted for over 24 hours into another hospital, received oral anti-malarials in last week, received oral antibiotics in last week, received traditional medicine in last week: n(%) yes, no, don't know
- N**umber of doses of IV or IM quinine/artesunate received before enrolment: median (IQR) or tabulation depending on numbers**

Past clinical history

- Two or more hospital admissions in the last year, previously received a blood transfusion (ever), received anti-helminths in last 6 months, has epilepsy, able to sit unsupported before this illness, able to walk without help before this illness: n(%) yes, no, don't know
- On regular anticonvulsants (n % of those with epilepsy) yes, no, don't know.

Child's family

- Number of siblings: median (IQR), range
- Any siblings with sickle cell disease: n(% of those with siblings) yes, no, don't know
- Father's ethnic group, mother's ethnic group
- Mother attended secondary school, child sleeps under a bed net/mosquito net: n(%) yes, no, don't know
- Parents alive: n(%) both alive, one alive, both dead
- Child's homestead: n(%) urban, semi-urban, rural

Clinical examination

- In-drawing, deep breathing, sunken eyes, decreased skin turgor, cold hands or feet only, liver size>2cm below costal margin, jaundice, very severe wasting/marasmus, generalised lymphadenopathy, flaky paint dermatitis, oral candidasis: n(%) yes, no, not assessed
- Crackles: n(%) unilateral, bilateral, none, not assessed
- Splenomegaly: n(%) not palpable, enlarged, gross

Docusign Envelope ID: AB5F25C1-23D2-4CE0-B871-54E4507AD149

- Signs of kwashiorkor (oedema): n(%) none, pretibial (minimum), hands/legs (moderate), generalised (severe)

Neurological

- Inability to sit up right unsupported, fitting currently, neck stiffness: n(%) yes, no, not assessed
- Bulging fontanelle (infants only) : n(% infants) yes, no, not assessed
- Pupil symmetry: n(%) equal, unequal

Ward tests at admission

- HIV test result: n(%) previously positive, previously negative, tested positive today, tested negative today, tested today invalid
- **Lactate (mmol/l), glucose (mmol/l): median (IQR)**
- **Glucose given: n (%) yes, no,**
- **Blood gas: pH, bicarb, PCO_2, Base deficit (ecf), base deficit (b): median (IQR)**

Presentation

- Healthcare facility first presented to: n(%) this hospital, level II, level III, level IV, other district hospital, private hospital
- Time to enrolment since presented at other facility, time to enrolment since referred from other facility: median (IQR), range
- Distance from other facility (estimated km): median (IQR), range

Admission blood test results and admission microbiology

- **Malaria RDT test: n(%) positive, negative, invalid or not done**
- **Malaria blood film: n(%) positive, negative, invalid or not done**
- Malaria pigment: n(%) yes, no
- Malaria species: n(% those with malaria) *P. falciparum*, *P. malariae*, *P. ovale*, *P. vivax*
- Parasite count per 200 WBC, parasite count per 500 RBC: median (IQR)
- WBC, RBC, Hb from FBC, haematocrit, MCV, MCH, MCHC, platelets, lymphocytes, neutrophils, granulocytes, monocytes, reticulocyte count: median (IQR)
- Sodium, potassium, urea/BUN, creatinine, albumin, AST, ALT, bilirubin: median (IQR)
- Pathogens isolated: n(% samples tested) yes, no.
- List of pathogens: n(%)

Description of follow-up

Denominator in each case is those who have been enrolled long enough ago for that visit to have occurred or to have completed follow up as appropriate, including those who have been lost to follow up.

Completion of follow up visits

- **Visits considered complete, defined as attended or died before the visit took place, at 7 days, at 28 days,** and overall**: n(%)**

Docusign Envelope ID: AB5F25C1-23D2-4CE0-B871-54E4507AD149

- **Child status at 7 day, 28 days,** and overall**: n(%) visit done, died, lost to follow up, missed visit.**

Co**mpleteness of neurological assessment records**

- **Records considered complete, defined as a non-missing entry or died before the time point at 28 days: n(%)**

Completeness of safety and efficacy data at 8 hours and 24 hours

- **Records considered complete, defined as a non-missing entry or died before the time point: n(%)**

Adherence to treatment

Number received infusion at 0 hours: N (%)
Number received infusion at 8 hours: N (%)
Number received infusion at 16 hours: N (%)

Primary analysis

Number of APTT>2.5xULN 1h post any sevuparin dose (grade 3 following the CTC) at each dose level: N(%)

Secondary analysis

Efficacy

Change in lactate from 0 to 8 hours: mean (sd) overall and by each dose level
Presence of mature infected erythrocytes on the blood films at 8 and 24 hours: N (%) overall and by each dose level
Parasite clearance time: mean (sd) hrs overall and by each dose level
If available: Change in sublingual microcirculation over time: mean (sd) proportion of perfused vessels; mean (sd) average perfusion speed indicator overall and by each dose level calculated using a Capillary Network Analysis.

Safety Endpoints

APTT 24h post enrolment (absolute level and grade): mean (sd) and N (%) with each grade, overall and by each dose level.
Neurological sequelae present at day 28: N(%)
Died by day 28: N(%)
Serious adverse events by day 28: N(%)

Docusign Envelope ID: AB5F25C1-23D2-4CE0-B871-54E4507AD149

Grade 3/4 adverse events by day 28: N(%)
