# Supplementary material for: Sevuparin in children with severe malaria: a phase I safety and dose-finding trial
Source: Nat Commun. 2026 Oct 21;17:10693. doi: 10.1038/s41467-026-78536-3 (PMC13686957; doi:10.1038/s41467-026-78536-3)
Supplement: Supplementary file 2 [file 41467_2026_78536_MOESM2_ESM.pdf]

nature portfolio

Corresponding author(s): Kathryn Maitland

Last updated by author(s): Aug 4, 2026

Reporting Summary

Nature Portfolio wishes to improve the reproducibility of the work that we publish. This form provides structure for consistency and transparency in reporting. For further information on Nature Portfolio policies, see our Editorial Policies and the Editorial Policy Checklist.

Statistics

For all statistical analyses, confirm that the following items are present in the figure legend, table legend, main text, or Methods section.

n/a	Confirmed	
☐	☒	The exact sample size (*n*) for each experimental group/condition, given as a discrete number and unit of measurement
☐	☒	A statement on whether measurements were taken from distinct samples or whether the same sample was measured repeatedly
☒	☐	The statistical test(s) used AND whether they are one- or two-sided<br>*Only common tests should be described solely by name; describe more complex techniques in the Methods section.*
☒	☐	A description of all covariates tested
☒	☐	A description of any assumptions or corrections, such as tests of normality and adjustment for multiple comparisons
☐	☒	A full description of the statistical parameters including central tendency (e.g. means) or other basic estimates (e.g. regression coefficient) AND variation (e.g. standard deviation) or associated estimates of uncertainty (e.g. confidence intervals)
☒	☐	For null hypothesis testing, the test statistic (e.g. *F*, *t*, *r*) with confidence intervals, effect sizes, degrees of freedom and *P* value noted<br>*Give P values as exact values whenever suitable.*
☒	☐	For Bayesian analysis, information on the choice of priors and Markov chain Monte Carlo settings
☒	☐	For hierarchical and complex designs, identification of the appropriate level for tests and full reporting of outcomes
☒	☐	Estimates of effect sizes (e.g. Cohen's *d*, Pearson's *r*), indicating how they were calculated

Our web collection on statistics for biologists contains articles on many of the points above.

Software and code

Policy information about availability of computer code

Data collection	Data was entered into an Open Clinica database (v3.13) hosted by the Kemri Wellcome Clinical Trial Facility, Kenya.
Data analysis	Data was extracted from the database in .csv files and analysed using Stata v19.1.

For manuscripts utilizing custom algorithms or software that are central to the research but not yet described in published literature, software must be made available to editors and reviewers. We strongly encourage code deposition in a community repository (e.g. GitHub). See the Nature Portfolio guidelines for submitting code & software for further information.

Data

Policy information about availability of data

All manuscripts must include a data availability statement. This statement should provide the following information, where applicable:

- Accession codes, unique identifiers, or web links for publicly available datasets
- A description of any restrictions on data availability
- For clinical datasets or third party data, please ensure that the statement adheres to our policy

Data are available through the online repository for KEMRI–Wellcome Trust Research Programme: Harvard Dataverse. Individual-level data, excluding personal identifiers, will be made available in accordance with an application to the Data Governance Committee, which meets monthly (dgc@kemri-wellcome.org). There are no timing restrictions on the availability of data. Source data for figures are provided with this paper.

Research involving human participants, their data, or biological material

Policy information about studies with human participants or human data. See also policy information about sex, gender (identity/presentation), and sexual orientation and race, ethnicity and racism.

Reporting on sex and gender	Sex is reported but not gender.
Reporting on race, ethnicity, or other socially relevant groupings	Kenyan and Zambian children were enrolled in this study. Data on ethnic background was not collected
Population characteristics	This was a single arm study enrolling eligible children presenting to hospital with severe malaria. The median age was 35 months (IQR 24, 48) and 12 (60%) were female. The range of severity features (coma, lactic acidosis, anaemia) are typical of the complications of paediatric severe malaria.
Recruitment	Eligible children were recruited at admission to the two hospitals based on the strict enrollment criteria and parental consent. Screening all potentially eligible children was continuous during enrollment periods thus bias in selection is unlikely.
Ethics oversight	Ethical approval was obtained from Imperial College Research Ethics Committee (ICREC) on 13th July 2021 (protocol number 18IC4513), Kenya Scientific and Ethics Review Unit (SERU), Nairobi Kenya on 25th February 2019 (protocol number GCMR-C/127/3744), Imperial College Research Ethics Committee (ICREC) on 13th July 2021 (protocol number 18IC4513) and the Tropical Diseases Research Centre Ethics Review Committee Ndola, Zambia and National Health Research Ethics Board, Lusaka, Zambia on 24th July 2023 (protocol number NHREB001/24/07/2023). This is reported in the study methods

Note that full information on the approval of the study protocol must also be provided in the manuscript.

Field-specific reporting

Please select the one below that is the best fit for your research. If you are not sure, read the appropriate sections before making your selection.

☒ Life sciences ☐ Behavioural & social sciences ☐ Ecological, evolutionary & environmental sciences

For a reference copy of the document with all sections, see nature.com/documents/nr-reporting-summary-flat.pdf

Life sciences study design

All studies must disclose on these points even when the disclosure is negative.

Sample size	23
Data exclusions	3 patients enrolled in Kilifi, Kenya did not have APPT times measures and therefore were excluded from the analysis of the primary endpoint
Replication	*Describe the measures taken to verify the reproducibility of the experimental findings. If all attempts at replication were successful, confirm this OR if there are any findings that were not replicated or cannot be reproduced, note this and describe why.*
Randomization	By design the children enrolled and dose allocated by continual reassessment method (to identify the maximum tolerated dose, MTD). The dose-toxicity curve was then re-fitted to the data after each cohort of 2 children was enrolled, based on their observed dose and whether they had a dose limiting toxicity event. After the first 4 children, each subsequent cohort was assigned the next dose based on the model's recommendation confirmed by an independent monitoring committee.
Blinding	The clinical staff were not blind to allocated dose. The laboratory staff measuring the APPT levels were blinded to dose.

Reporting for specific materials, systems and methods

We require information from authors about some types of materials, experimental systems and methods used in many studies. Here, indicate whether each material, system or method listed is relevant to your study. If you are not sure if a list item applies to your research, read the appropriate section before selecting a response.

Materials & experimental systems

n/a	Involved in the study	
☒	☐	Antibodies
☒	☐	Eukaryotic cell lines
☒	☐	Palaeontology and archaeology
☒	☐	Animals and other organisms
☐	☒	Clinical data
☒	☐	Dual use research of concern
☒	☐	Plants

Methods

n/a	Involved in the study	
☒	☐	ChIP-seq
☒	☐	Flow cytometry
☒	☐	MRI-based neuroimaging

Clinical data

Policy information about clinical studies

All manuscripts should comply with the ICMJE guidelines for publication of clinical research and a completed CONSORT checklist must be included with all submissions.

Clinical trial registration	Trial registration ISRCTN32271864 (registered on 28/07/2021) and PACTR202007890194806 (registered on 20/07/2020)
Study protocol	The trial protocol has been published. DOI 10.12688/wellcomeopenres.20111.2 It is open access.
Data collection	Clinical and laboratory data were collected in Kilifi County Hospital, Kilifi, Kenya and St Pauls Mission Hospital, Nchelenge, Luapula Province, Zambia on paper CRFs and entered into an Open Clinica database. The children were enrolled between 1st September 2022 and 5th March 2025, 23 children in Kenya and Zambia were enrolled and each child followed up to Day 28 (last participant last visit on 4th April 2025).
Outcomes	The primary outcome was development of a DLT defined as APTT>2.5 x ULN (grade 3 following the Common Toxicity Criteria v5.032) translating to > 95 seconds (assuming a normal range of 21-38 seconds) 1 hour post sevuparin dose. Secondary outcomes change in lactate from 0 to 8 hours; presence of mature infected erythrocytes on the blood films at 8 and 24 hours, and parasite clearance time. Safety endpoints included APTT 24h post enrolment (absolute level and grade), development of abnormalities of coagulation indices (prothrombin) (Grade ≥2), neurological sequelae through day

Plants

Seed stocks	*Report on the source of all seed stocks or other plant material used. If applicable, state the seed stock centre and catalogue number. If plant specimens were collected from the field, describe the collection location, date and sampling procedures.*
Novel plant genotypes	*Describe the methods by which all novel plant genotypes were produced. This includes those generated by transgenic approaches, gene editing, chemical/radiation-based mutagenesis and hybridization. For transgenic lines, describe the transformation method, the number of independent lines analyzed and the generation upon which experiments were performed. For gene-edited lines, describe the editor used, the endogenous sequence targeted for editing, the targeting guide RNA sequence (if applicable) and how the editor was applied.*
Authentication	*Describe any authentication procedures for each seed stock used or novel genotype generated. Describe any experiments used to assess the effect of a mutation and, where applicable, how potential secondary effects (e.g. second site T-DNA insertions, mosiacism, off-target gene editing) were examined.*
