# Supplementary material for: Sevuparin in children with severe malaria: a phase I safety and dose-finding trial
Source: Nat Commun. 2026 Oct 21;17:10693. doi: 10.1038/s41467-026-78536-3 (PMC13686957; doi:10.1038/s41467-026-78536-3)
Supplement: Supplementary file 3 [file 41467_2026_78536_MOESM3_ESM.pdf]

nature portfolio

Peer Review File

Sevuparin in children with severe malaria: A phase I safety and dose finding trial

Corresponding Author: Professor Kathryn Maitland

This file contains all reviewer reports in order by version, followed by all author rebuttals in order by version.

Version 0:

Reviewer comments:

Reviewer #1

(Remarks to the Author)
In this Phase I, open-label, dose-finding trial, Chaponda and colleagues used a continual reassessment method to identify the maximum tolerated dose of a three-dose intravenous sevuparin regimen, administered as an adjunct to intravenous artesunate for the treatment of severe malaria. Based on data from 20 participants with APTT measurements obtained one hour after drug administration, the authors identified a maximum tolerated dose of 3 mg/kg, consistent with doses previously evaluated in adults with severe malaria in Asia. Three children were enrolled but did not have post-dose APTT measurements available. Overall, the trial appears to have been well conducted, with appropriate safety monitoring, and the study conduct appears consistent with the protocol and statistical analysis plan.

I have two points for consideration.

First, given that the trial was conducted in the context of intravenous artesunate associated with rapid parasite clearance, the rationale for timing of parasite clearance sampling should be justified. In particular, the authors should explain why this sampling strategy was chosen, given that the approach does not fully align with WWARN guidance for parasite clearance estimation.

Second, data on the secondary efficacy endpoint, changes in sublingual microcirculation, were not included in the reported results. Given the mechanism of action of Sevuparin in reducing sequestration of mature parasites to improve microvascular function, the rationale for not including this pre-defined outcome should be discussed and acknowledged as a limitation.

Reviewer #2

(Remarks to the Author)
Chaponda et al. present the findings of a dose-finding trial of a heparin-like compound, Sevuparin, aimed to identify the maximum tolerated dose (MTD) in children with severe malaria. Primary outcome was safety, assessed as dose limiting toxicity defined as an APTT >95sec. They identified the MTD that can be used for future Phase II efficacy studies on the added benefit of Sevuparin on the treatment of severe malaria.

Overall the study is methodologically well designed and carefully excecuted. It represents a necessary step forward in the identification of possible new adjunctive treatments for severe malaria in children, which are still sorely needed.

Since this study had no control group, a limitation already acknowledged by the authors, I would be careful with certain phrasing in the discussion. As stated in the methods, lactate clearance at 8 hours was assessed as a secondary objective to evaluate its feasibility as a future Phase II endpoint. As currently phrased (line 188), I would argue this is more presented as an efficacy finding, I would suggest rephrasing this sentence to fit more within the outlined study objectives. The subsequent comparison of mortality in this study to baseline mortality risk and mortality rates found in observational studies also frames the differences as a possible effect of Sevuparin. However trial participation itself is associated with increased monitoring and clinical attention, which cannot be ruled out as a contributing factor given the absence of a control group. Comparisons to baseline risk and observational data should therefore be interpreted with caution and could warrant a sentence acknowledging this limitation where the comparison is made.

I have outlined several other suggestions and comments below.

Editing comments:

Repetition of both (line 76)

Consequent use of italics throughout manuscript: in vitro (line 86), a priori (line 251)

"Testing this hypothesis" is a dangling participle here, since the sentence's grammatical subject is the "it" after the comma, which cannot logically perform the action of testing. Suggest replacing with the infinitive of purpose: "to test this hypothesis." (line 116)

The difference between primary and intention to treat analysis becomes a bit unclear in the results paragraph: safety and other exploratory endpoints, and the beginning of the discussion.
In line 168 the safety analysis of the intention to treat population is introduced, stating that this includes two more SAEs compared to the primary analysis group: 1 death and 1 readmission due to anemia. However in the next paragraph it states that in the analysis of all children (without APTT measures, which is the intention to treat population already introduced?) there is only one additional SAE: readmission due to convulsions?
According to Table S3 and Table 2 the death occurred in the primary population and only one additional non-fatal SAE occurred in the intention to treat population. Please align the text with the numbers in the Tables.
Depending on changes made in the results, also adjust the information in line 183, 184 and 185 of the discussion (the amount of SAEs).

Figures and Tables:

Figure S3 is missing from appendix (line 196)

The number of participants stated in Figure 1 are incorrect. Currently, the same amount of participants screened (85), are also excluded.

Additional information:

It might be worthwhile to add the recruitment site per participant in the baseline characteristics table, since the transmission intensities between the sites are quite different and this can influence the clinical presentation or even the definition of severe malaria, baseline parasitemia etc.

Since safety is the main priority, it might be worthwhile to add a figure on thrombocyte count during treatment (if available), since it is possible that this declines further after inclusion and can cause or excacerbate bleeding in combination with Sevuparin.

Reviewer #3

(Remarks to the Author)
I had the pleasure of reviewing the manuscript, “Sevuparin in children with severe malaria: A phase I safety and dose-finding trial.”
The study assessed the maximum tolerated dose of sevuparin as an adjunctive treatment for severe malaria. This phase 1 trial is timely, as mortality from severe malaria remains high despite the availability of highly effective antimalarial treatments. The manuscript is well written; the analytic plan and discussion of the results are clear. I have a few comments that I would like the authors to address before publication.

1. The eligibility criteria appear to have selected children with cerebral malaria, lactic acidosis, and severe anaemia—syndromes that are likely to be associated with greater parasite sequestration. If this was intentional, the authors should explain why acute kidney injury was not included, given that it is increasingly reported and is associated with high fatality in severe malaria. In addition, it would be helpful to clarify whether children were required to have only one severe malaria complication, or whether children with overlapping complications—for example, cerebral malaria with severe anaemia or lactic acidosis—were eligible, as commonly occurs in paediatric severe malaria.

2. Although the study was conducted in a scientifically sound manner, the absence of baseline measurements for some children—such as lactate levels, blood smears to estimate parasite clearance time as per WWRAN and APTT—resulting in the exclusion of three children from the final analysis is concerning, even for a dose-finding study that was not necessarily powered to assess efficacy. Was there a particular reason why these were missed for such a small sample sized study?

3. The results are presented in a clear manner, and the authors are commended for a great job and I look forward to the phase II efficacy trial

Open Access This Peer Review File is licensed under a Creative Commons Attribution 4.0 International License, which permits use, sharing, adaptation, distribution and reproduction in any medium or format, as long as you give appropriate credit to the original author(s) and the source, provide a link to the Creative Commons license, and indicate if changes were made.
In cases where reviewers are anonymous, credit should be given to 'Anonymous Referee' and the source.
The images or other third party material in this Peer Review File are included in the article's Creative Commons license, unless indicated otherwise in a credit line to the material. If material is not included in the article's Creative Commons license and your intended use is not permitted by statutory regulation or exceeds the permitted use, you will need to obtain permission directly from the copyright holder.
To view a copy of this license, visit https://creativecommons.org/licenses/by/4.0/

Response to reviewers

We thank all reviewers for their careful consideration of the manuscript and very helpful comments for clarification and suggested additions to the report. We respond point by point as follows.

Reviewer 1

1) *Given that the trial was conducted in the context of intravenous artesunate associated with rapid parasite clearance, the rationale for timing of parasite clearance sampling should be justified. In particular, the authors should explain why this sampling strategy was chosen, given that the approach does not fully align with WWARN guidance for parasite clearance estimation*.

The reviewer rightly notes that artesunate results in rapid clearance of malaria parasites, which may mask any early effects of sevuparin on clearance. Whilst we agree that the selected timepoints were sub-optimal and did not align with WWARN guidance for parasite clearance estimation, assessing parasite clearance was not a primary objective of the trial, which was a single-arm non-comparative study focussing on identifying an optimal sevuparin dose based on dose-limiting toxicities using the continuous reassessment method. Therefore, the sampling timepoints were aligned with those required for clinical care, and were specifically designed to avoid too many blood sampling timepoints in these critically ill children, given the need to take samples for pharmacokinetic analysis. We have indicated this as a limitation in the Discussion and clarified that additional timepoints to estimate parasite clearance more accurately would need to be incorporated in any future efficacy investigation.

On the topic of parasite clearance, we have also added some discussion on the findings of the Study TSM02 (reference 27 in the original submission, now 26). This was a phase II study in uncomplicated *P. falciparum* malaria in adults in which the intervention (sevuparin 6 hourly) was given alongside oral atovaquone and 400mg proguanil (Malanil) and compared to Malanil alone (control)[1]. Following the initial infusion of sevuparin, an immediate effect was observed on the (early) ring-stage infected erythrocytes which decreased consistently for six hours, whereas in the control group the parasites continued to expand in the controls (Figure 4A in the published article). This indicates a direct effect of sevuparin on parasite clearance prior to therapeutic levels of artemisinin, now noted in the Discussion.

2) *Data on the secondary efficacy endpoint, changes in sublingual microcirculation, were not included in the reported results. Given the mechanism of action of Sevuparin in reducing sequestration of mature parasites to improve microvascular function, the rationale for not including this pre-defined outcome should be discussed and acknowledged as a limitation.*

The team in Kilifi had received training on the side stream-dark field imaging (using CytoCam, Braedius Medical BV®) pre-COVID in early 2020 whilst the protocol was under ethics review. The COVID epidemic led to a very delayed protocol approval and trial initiation. Unfortunately, by the time the study finally started, the CytoCam was no longer functional. We have included the reason why this was not measured in the trial limitations section in the Discussion and have indicated that any future investigation of sublingual microcirculation would provide supportive physiological data, and that would be its role rather than as a secondary efficacy endpoint.

Reviewer 2

Since this study had no control group, a limitation already acknowledged by the authors, I would be careful with certain phrasing in the discussion.

1) *As stated in the methods, lactate clearance at 8 hours was assessed as a secondary objective to evaluate its feasibility as a future Phase IIb endpoint. As currently phrased (line 188), I would argue this is more presented as an efficacy finding, I would suggest rephrasing this sentence to fit more within the outlined study objectives.*

We thank the reviewer for highlighting this and have modified the sentence in the Discussion to align the reporting with the study objectives. We have also added the secondary objective to the last sentence of the Introduction, since otherwise the Methods which refers to this secondary objective comes after its first mention in the Discussion.

2) *The subsequent comparison of mortality in this study to baseline mortality risk and mortality rates found in observational studies also frames the differences as a possible effect of Sevuparin. However trial participation itself is associated with increased monitoring and clinical attention, which cannot be ruled out as a contributing factor given the absence of a control group. Comparisons to baseline risk and observational data should therefore be interpreted with caution and could warrant a sentence acknowledging this limitation where the comparison is made.*

We fully understand the reviewer's concerns given that this was a small clinical trial with rigorous monitoring which may have improved the detection of clinical complications (i.e. adverse events which may not be detected in usual clinical care). We have therefore incorporated this caution into the discussion, as a caveat. However, we would note that the same clinical teams were involved in both the observational study (ISRCTN14711763) referred to in the Discussion and this Phase I trial, and these teams provided additional support for monitoring and clinical care in both the two studies. We note that across most studies mortality in cerebral malaria (~20-25%) has largely remained unchanged over the decades[2,3], and even remained high in children with coma enrolled in the AQUAMAT trial (who also received a higher level of monitoring/clinical supportive care): artesunate vs quinine arm (18% vs 21%)[4].

Editing comments:
Repetition of both (line 76)#

This has been removed

Consequent use of italics throughout manuscript: in vitro (line 86), a priori (line 251)

Now italicized

"*Testing this hypothesis" is a dangling participle here, since the sentence's grammatical subject is the "it" after the comma, which cannot logically perform the action of testing. Suggest replacing with the infinitive of purpose: "to test this hypothesis." (line 116)*

We have amended to use the reviewer's suggestion 'To test this hypothesis'

The difference between primary and intention to treat analysis becomes a bit unclear in the results paragraph: safety and other exploratory endpoints, and the beginning of the discussion. In line 168 the safety analysis of the intention to treat population is introduced, stating that this includes two more SAEs compared to the primary analysis group: 1 death and 1 readmission due to anemia. However, in the next paragraph it states that in the analysis of all children (without APTT measures, which is the intention to treat population already introduced?) there is only one additional SAE: readmission due to convulsions?

We have edited the results section to make it clearer that the 1 death and 1 readmission were in the primary analysis, and then in the analysis of all children there is one additional readmission.

According to Table S3 and Table 2 the death occurred in the primary population and only one additional non-fatal SAE occurred in the intention to treat population. Please align the text with the numbers in the Tables.

Clarified as above.

Depending on changes made in the results, also adjust the information in line 183, 184 and185 of the discussion (the number of SAEs).

This has now been corrected.

Figures and Tables:

# Figure S3 is missing from appendix (line 196)#

In the final edit we removed this Figure so have deleted this in the manuscript.

The number of participants stated in Figure 1 are incorrect. Currently, the same amount of participants screened (85), are also excluded.

This has now been corrected.

Additional information:

# It might be worthwhile to add the recruitment site per participant in the baseline characteristics table, since the transmission intensities between the sites are quite different and this can influence the clinical presentation or even the definition of severe malaria, baseline parasitemia etc.

All 20 children included in the primary analysis were from Zambia (added to main Results text and Table 1 footnote). We have also added the site for all children in Table S1 (Kenya plus Zambia) which shows a similar proportion of children with complications to Table 1 (Zambia alone), indicating that the addition of the three children recruited at Kenya to the secondary analysis population does not impact the clinical definition.

# Since safety is the main priority, it might be worthwhile to add a figure on thrombocyte count during treatment (if available), since it is possible that this declines further after inclusion and can cause or exacerbate bleeding in combination with Sevuparin.

We did not record this parameter beyond baseline, so are unfortunately unable to add this.

Reviewer 3

I had the pleasure of reviewing the manuscript, “Sevuparin in children with severe malaria: A phase I safety and dose-finding trial.”
The study assessed the maximum tolerated dose of sevuparin as an adjunctive treatment for severe malaria. This phase 1 trial is timely, as mortality from severe malaria remains high despite the availability of highly effective antimalarial treatments. The manuscript is well written; the analytic plan and discussion of the results are clear. I have a few comments that I would like the authors to address before publication.

1. The eligibility criteria appear to have selected children with cerebral malaria, lactic acidosis, and severe anaemia—syndromes that are likely to be associated with greater parasite sequestration. If this was intentional, the authors should explain why acute kidney injury was not included, given that it is increasingly reported and is associated with high fatality in severe malaria. In addition, it would be helpful to clarify whether children were required to have only one severe malaria complication, or whether children with overlapping complications—for example, cerebral malaria with severe anaemia or lactic acidosis—were eligible, as commonly occurs in paediatric severe malaria.

The eligibility criteria were based on selected complications from the current WHO definition of severe malaria plus a high lactate at baseline – the latter ensured we were able to investigate a reduction in lactate as a secondary endpoint. We did not include a criteria based on anaemia:

further various studies have shown that anaemia does not necessarily increase malaria-associated mortality unless profound: only 15% of the primary population had severe anaemia (Hb<4g/dl). All children needed to have lactic acidosis. Children with overlapping complications (coma and deep breathing for example) were eligible, they just needed one criteria as a minimum to be enrolled. We did not record baseline creatinine or urea, hence these were not inclusion criteria. We appreciate that more data and evidence is coming to light around the importance of acute kidney injury in paediatric severe malaria; however, the trial was designed pre-COVID, and, as above, we wanted to minimise additional blood sampling (as may be needed for some definitions of acute kidney injury (AKI)) and felt we would enrol sufficiently with our criteria as defined. Enrolling children with AKI would definitely be considered for the future. We have edited the methods section to clarify these points.

2. Although the study was conducted in a scientifically sound manner, the absence of baseline measurements for some children—such as lactate levels, blood smears to estimate parasite clearance time as per WWRAN and APTT—resulting in the exclusion of three children from the final analysis is concerning, even for a dose-finding study that was not necessarily powered to assess efficacy. Was there a particular reason why these were missed for such a small sample sized study?

Baseline lactate and parasite density read from malaria slides was available on all participants. The paucity of timepoints for slide readings following baseline was due to them being aligned around clinical care sampling. This explanation has now been added to the discussion, also see response to Reviewer 1 above. In error, the study team in Kilifi used Istat cartridges which recorded Activated clotting time (ACT) directly from whole blood rather than directly measuring the Activated Partial Thromboplastin Time (APTT) from plasma. Although providing similar information, they are not identical, hence we did not use this data from the children enrolled in Kenya for the analysis of the primary endpoint.

3. The results are presented in a clear manner, and the authors are commended for a great job and I look forward to the phase II efficacy trial

Many thanks for this commendation.

References

1. Leitgeb, A.M. *et al.* Inhibition of merozoite invasion and transient de-sequestration by sevuparin in humans with Plasmodium falciparum malaria. *PLoS One* **12**, e0188754 (2017).
2. Akech, S. *et al.* The Clinical Profile of Severe Pediatric Malaria in an Area Targeted for Routine RTS,S/AS01 Malaria Vaccination in Western Kenya. *Clin Infect Dis* **71**, 372-380 (2020).
3. Njuguna, P. *et al.* Observational study: 27 years of severe malaria surveillance in Kilifi, Kenya. *BMC Med* **17**, 124 (2019).
4. Dondorp, A.M. *et al.* Artesunate versus quinine in the treatment of severe falciparum malaria in African children (AQUAMAT): an open-label, randomised trial. *Lancet* **376**, 1647-57 (2010).
